# Supplementary material for: MultiSite Assembly of Gateway Induced Clones (MAGIC): a flexible cloning toolbox for use in vertebrate model systems
Source: Development. 2025 Nov 13;152(22):dev204308. doi: 10.1242/dev.204308 (PMC12669975; doi:10.1242/dev.204308)
Supplement: Supplementary information [file develop-152-204308-s1.pdf]

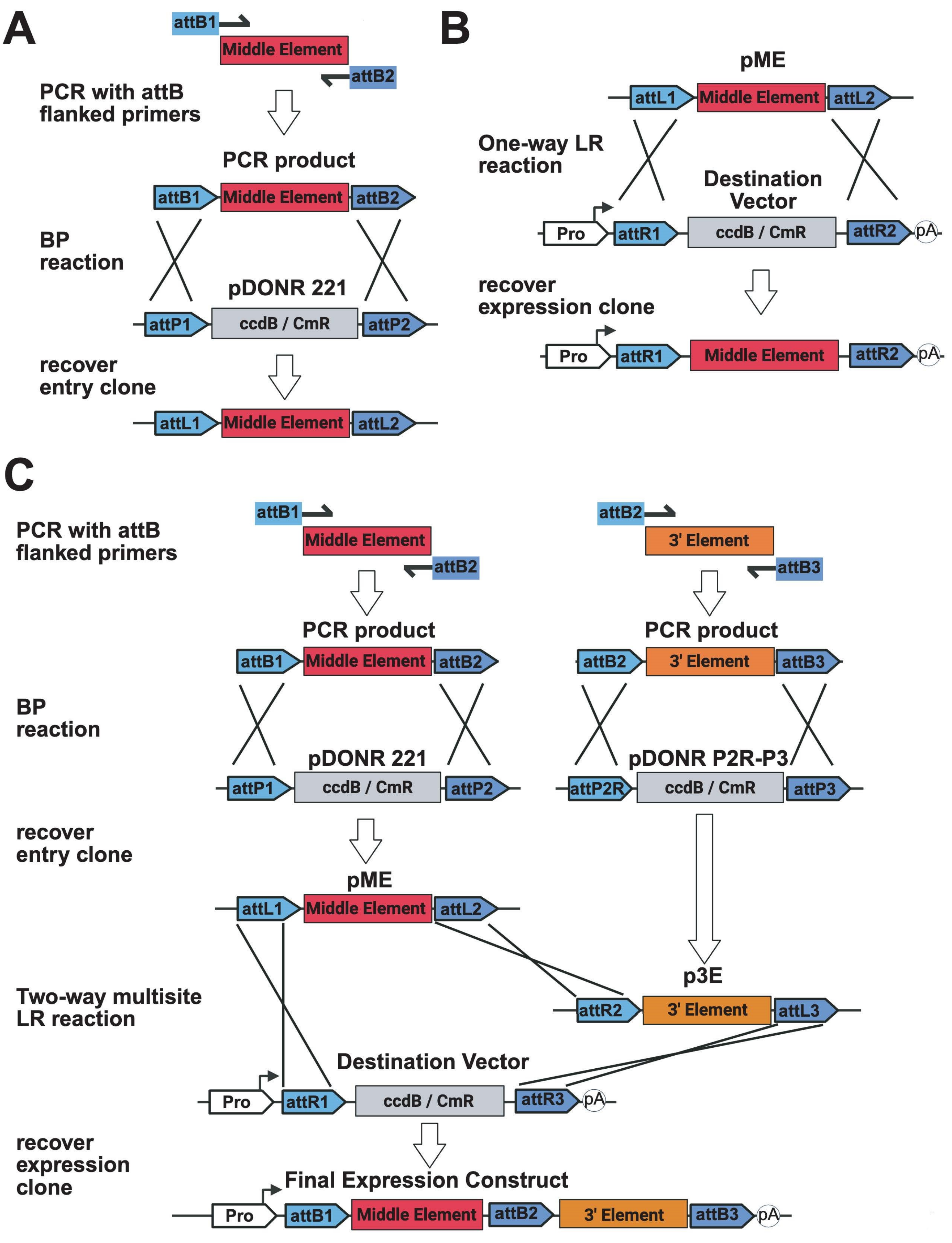

Fig. S1. Gateway recombination reactions. (A) Generation of an insert by adding flanking attB1/B2 arms via PCR and subsequent BP clonase-mediated recombination into a pDONR backbone to generate an ENTRY clone. (**B**) Diagram of a one-way LR recombination of an entry clone into an DESTination vector to generate an expression clone. (**C**) Examples of simultaneous generation of a middle entry attB1/B2 flanked insert and an attB2/B3 flanked 3' insert, both via PCR, and subsequent generation of a pME (middle ENTRY) and p3E (3' ENTRY) clones via BP clonase-mediated recombination, then a two-way LR reaction with a DEST plasmid to generate an expression clone containing both inserts.

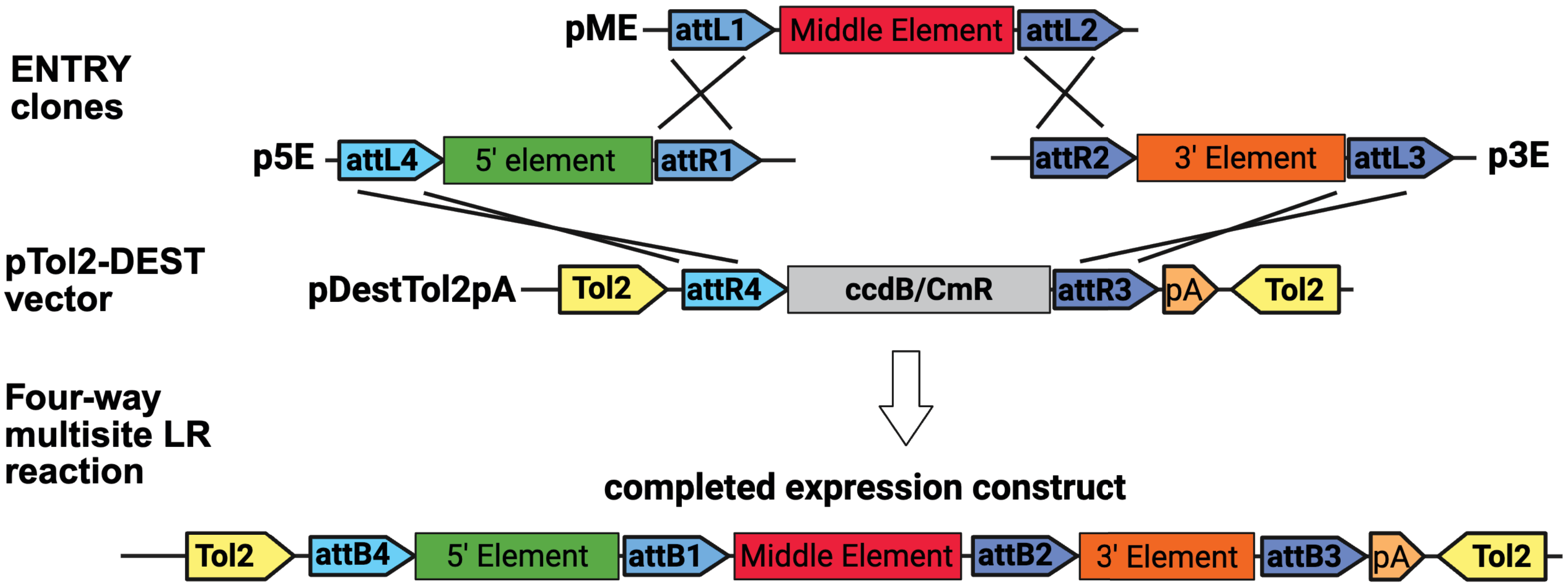

Fig. S2. The Logic of Three-way Gateway Recombination Cloning for the Zebrafish Tol2 System. (A) Schematic of a three-way LR reaction between an attL4/attR1 5' Entry clone (p5E), an attL1/attL2 middle entry clone (pME), and an attR2/attL3 3' entry clone (p3E) with a pTol2-DEST vector and the resultant expression vector.

Development • Supplementary information

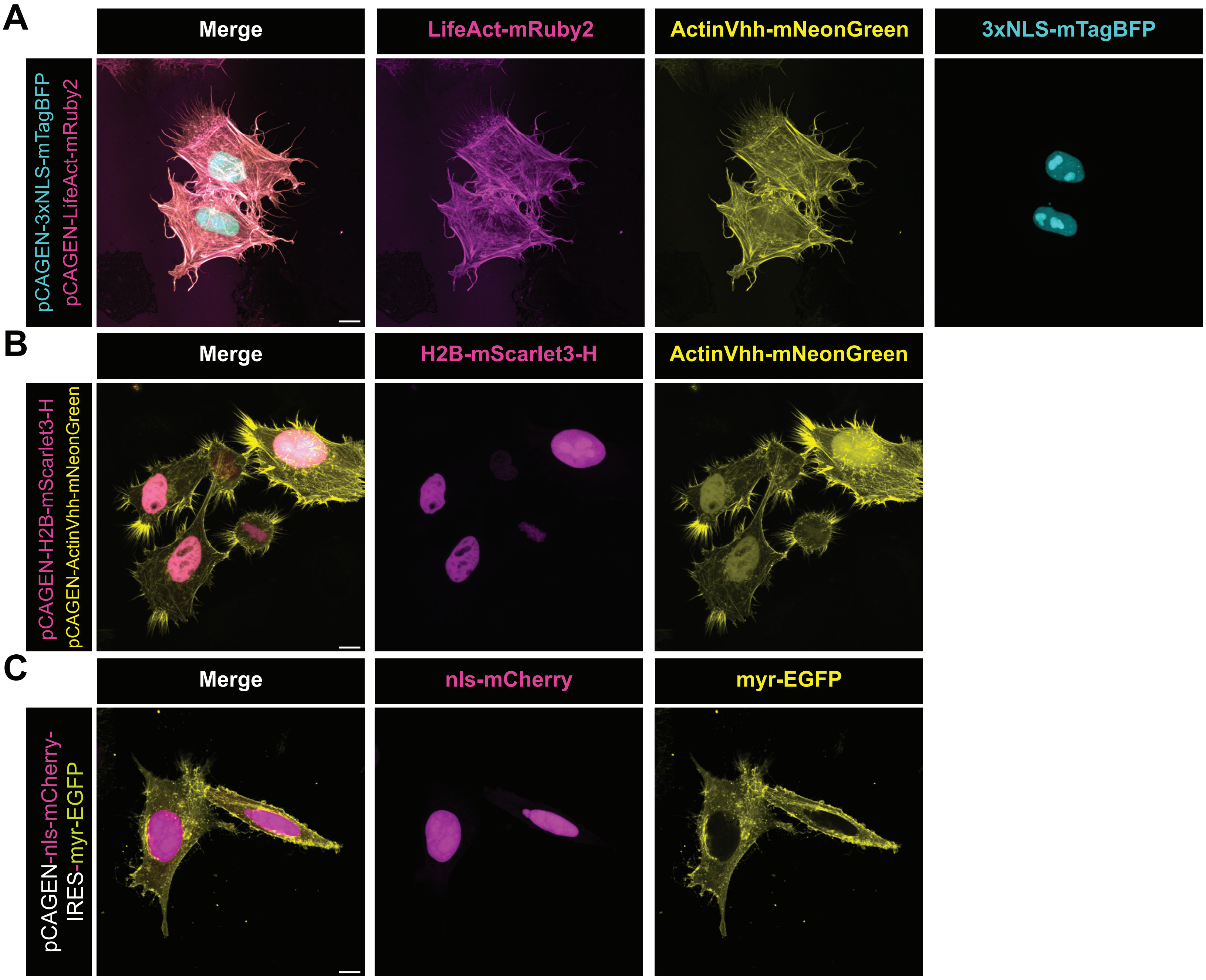

Fig. S3. In Vitro Validation of Novel Fluorescent Subcellular Reporters. Subcellular structures were labelled by transient transfection of fluorescent reporter clones generated from LR recombination reactions with pCAGEN-DEST and middle entry plasmids described in Table 3. **(A)** Nuclei are labelled with 3xNLS-mTagBFP2, while filamentous actin are labelled by a fusion of the F-actin probe, Lifeact, to mRuby2, and all actin is labelled using a pan-actin nanobody (actin vhh) fused to mNeonGreen. **(B)** Nuclei are labelled with an H2B fusion to mScarlet3-H while actin is labelled using a pan-actin nanobody (actin vhh) fused to mNeonGreen. (**C**) Nuclei are labelled with an NLS-mCherry reporter while the cell membrane is labelled via N-terminal myristoylation tag fused to EGFP. Scale bar = 10 µm.

Development • Supplementary information

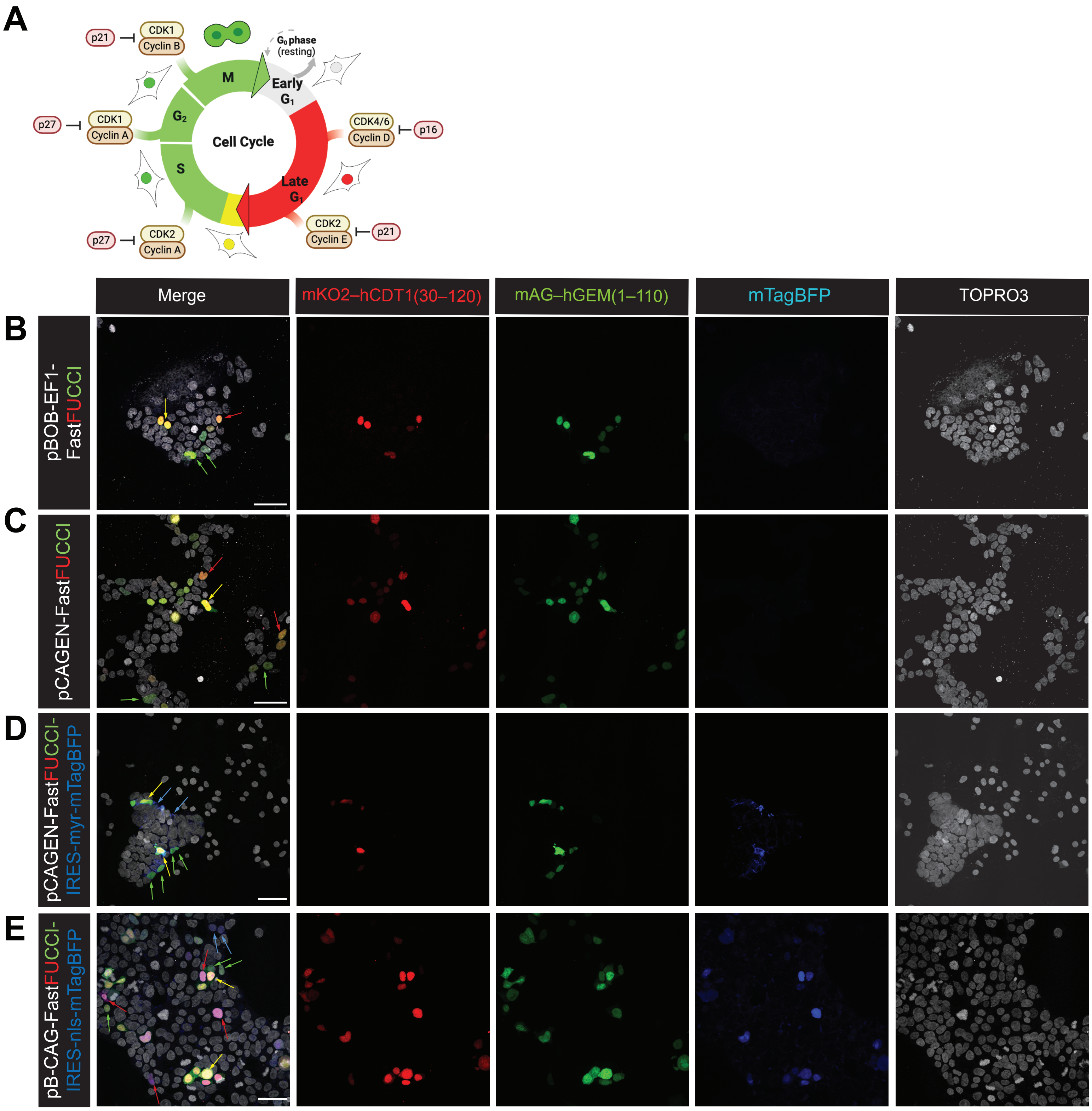

Fig. S4. In vitro Comparison of FastFucci Reporters. (A) Schematic of the FastFUCCI system originated by Koh and colleagues (Koh et al., 2017) where fusion of mKO2 to human CDT1 (amino acids 30-120) followed by a T2A peptide and mAG fused to human Geminin (amino acids 1-110) enables fluorescent visualization of progression through the mammalian cell cycle. (**B**) Transient transfection of the original pBOB-EF1a-FastFUCCI reporter into HEK-293 cells confirms activity of the reporter. (**C**) Recombination of pME-FastFUCCI into pCAGEN-DEST and transient transfection confirms the functionality of the middle entry clone. (**D**) LR recombination into pCAGEN-DEST-IRES-myr-mTagBFP and transient transfection confirms functionality of the cell cycle reporter and membrane localized mTagBFP to identity all transfected cells. (**E**) Multi-site recombination with p5E-CAG, pME-FastFUCCI, p3E-IRES-nls-mTagBFP and pB-DEST and transient transfection confirms functionality of the IRES-nlsBFP reporter in non FUCCI expressing cells. Red arrows denote cells in late G1, while yellow denotes cells transitioning from late G1 to S phase, and green denotes cells in S-G2-M phase. Blue denotes cells expressing the myr- or nls-BFP reporter. Scale bar = 50 μm. `Merge' images in C-E are from Fig. 2.

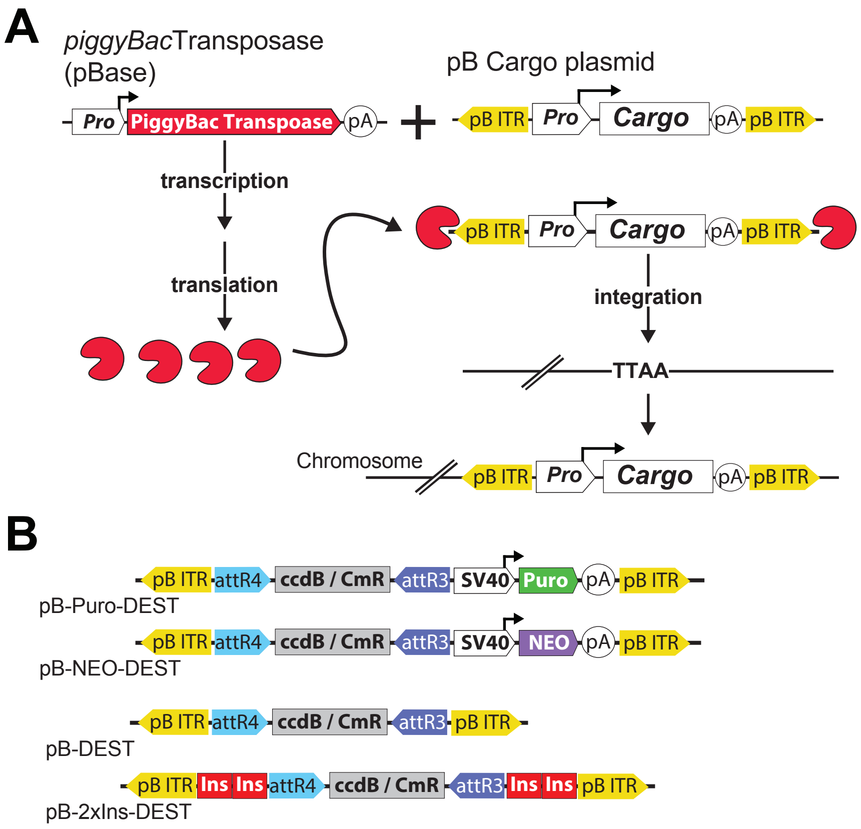

Fig. S5. *piggyBac* Transposase-Compatible Destination Vectors. (A) In the binary *piggyBac* system, *piggyBac* transposase promotes integration of a pB ITR flanked cargo cassette into the genome at AATT sequences. (**B**) A suite of selectable (i.e. Neomycin, Puromycin) and non-selectable pB ITR flanked Destination plasmids, with and without flanking insulator sequences to prevent positional effects upon genomic integration, for multisite Gateway cloning.

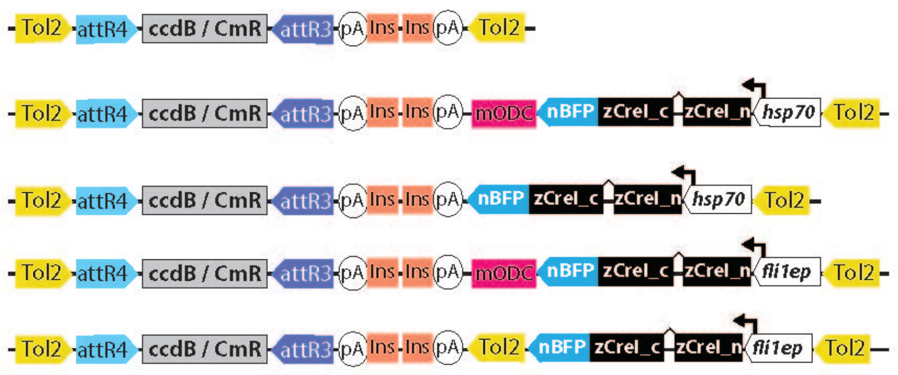

Fig. S6. New, All-in-One Destination Backbones for Tol2-mediated Transgenesis. A novel Tol2 flanked Destination vector with 2 insulator sequences separating the backbone from the Destination cassette (top). Below are other new derivative using the above backbone that include a heatshock-inducible promoter (*hsp70*) or endothelial-specific promoter (*fli1ep*) driving expression of Cre recombinase, codon optimized for expression in zebrafish (zCre) where the open reading frame is separated by an internal intron to prevent spurious expression and resultant loxP recombination in e. coli during routine cloning steps. Also provided are variants where zCre-BFP is fused to a murine Ornithine Decarboxylase (mODC) peptide to promote ubiquitination and turnover of the protein, preventing accumulation of Cre recombinase.

Development • Supplementary information

Plasmid Form | Plasmid List | Print Plasmid Label | Print Cell Stock Label

Wythe Lab Plasmid Details

Insert File

JDW 936 (pB_RAM_Flex_DEST_EFS_TetR)

Identification:

Name pB_Tet-Off_Flex_DEST_EFS_mODC_tTA

Number JDW 936 **Creator** Manuel Cantu Gutierrez

Use PiggyBac | Tet/Dox | Cre/Lox

PMID: **Addgene #** **MTA Needed?** **Sequence** CTAAATTGTAAGCGT

Date Sequenced 1/3/2020

Construction:

Promoter TRE-tight (7xTetop-CMVmin)

Insert DEST Cassette

Species **Size**

N-Tag **C-Tag**

2A/IRES/Fusion

Insert

Species **Size**

N-Tag **C-Tag**

Backbone JDW 880 (pB-pRAM-Flex-

Type Mammalian Expression

Promoter EFS (human)

Size 8299 bp

Reporter d2tTA

Growth:

Resistance AMP/CHLOR **Copy** High

Strain ccdB **Temp** 37C

Inventory:

DNA Stock Yes **E. Coli Stock** Yes

Comments and Cloning Details:

Piggyback DEST (destination) vector for pDONR221 (L1/L2) / pME middle entry clones where the DEST cassette is flanked by flexed WT and mutant loxP sites (i.e. "FLEX") for Cre-dependent, Tetracycline or Dox regulatable expression. This is a tTA (or **Tet-Off**) driven vector driven by an EF1a short or core promoter.
JDW 880 (pB-pRAM-Flex-mClover3-KrasG12D-WPRE-pA-EFS-TetR) was digested with NheI and AscI to add a DEST cassette. The DEST Cassette was amplified by PCR using:
oJDW 1398 -
oJDW 1399 -
The PCR insert was digested NheI and AscI and inserted into the backbone.

Map

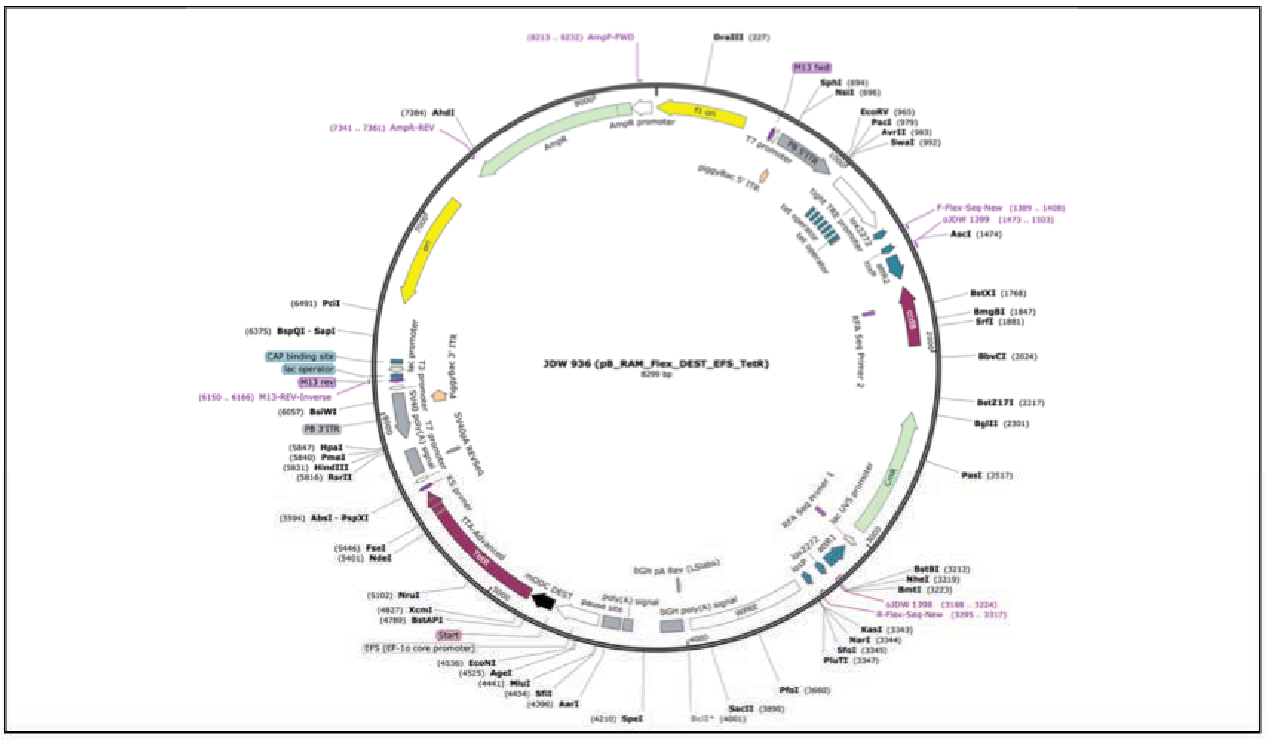

Fig. S7. A Typical Plasmid Record in the Plasmid Database. This format view shows an individual plasmid record, with several fields of important information. The first is at the top of the image in the Identification fields, where a user can assign a name and number for indexing purposes to each plasmid record, as well as other important criteria. Some examples of this include uses of the plasmid, such as *piggyBac* transgenesis, or Tet/Dox inducible systems, or Cre/lex, etc. Below, in the construction fields, one can indicate which promoter was used, what the insert is, species of the insert, and relevant tags or fusions, as well as the backbone, and any selectable markers, if applicable. Below, in the cloning and comments details, free text can be entered describing how the plasmid was generated and these details can help other users understand how the plasmid was made, and can also provide useful details if these details are required in a publication. Finally, the Growth and Inventory fields indicate the bacterial cell type, antibiotic resistance, temperature for optimal growth, as well as whether the DNA or glycerol stock is on hand in the lab. JPEG and PNG file format images can be inserted into the Map field, while APE and SNAPGENE or other format electronic files can be inserted into the Insert Field.

Development • Supplementary information

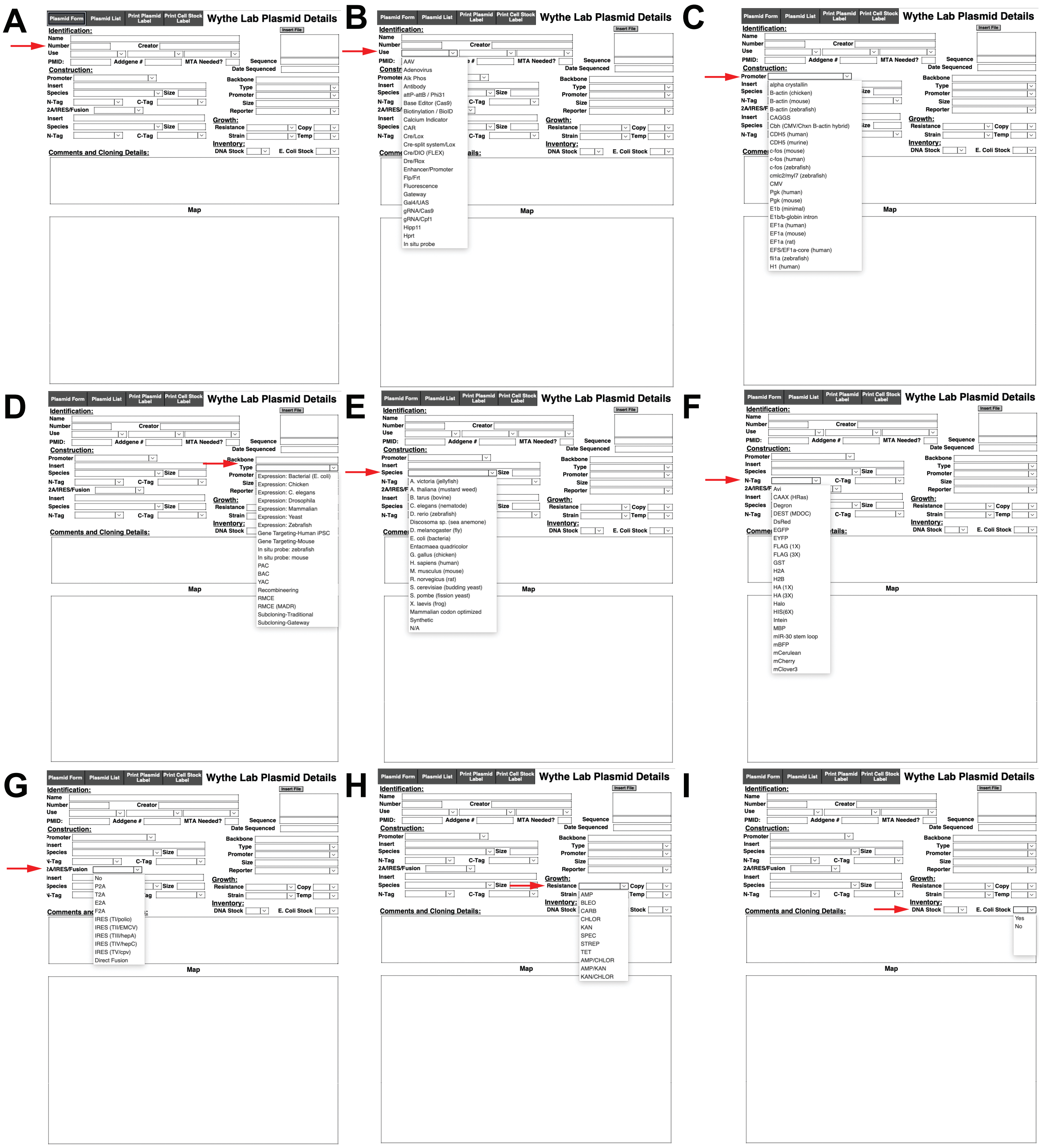

Development • Supplementary information

Fig. S8. Step by Step Data Entry for A New Plasmid Record. Moving from panel A through I, when a new record is created, a user should include the various details asked for to ensure a complete, searchable record is present for the new entry. **A)** We recommend first assigning a new plasmid number (ours go in sequence, such as JDW 1, JDW 2, etc.). **B)** Then, consider what use categories the new plasmid falls into using predefined terms. The advantage of a controlled vocabulary for the “use” category, as well as other categories in the database, ensures optimal results when searching your database for plasmids that meet specific criteria, rather than allowing users to enter various terms that may not be identical and could potentially be missed in searches. **C)** In the construction field, details, such as the “promoter” field, can be selected from a dropdown menu, or populated by manual text entry (again, we suggest altering the dropdown lists to reflect commonly used promoters in your research group to ensure better, more accurate search results). **D)** The “type” of plasmid is then selected, such as subcloning, or an expression plasmid, etc. **E)** Next, the insert and species of the insert (mammalian codon optimized, a human open reading frame or cDNA, etc. should be entered. **F)** If there are any C- or N-terminal tags, such as an NLS or an epitope tag, should be indicated. **G)** If there is a second insert via a direct fusion, or 2A cleavage peptide sequence, or an IRES should be indicated. **H)** Information such as antibiotic resistance, copy number, and more can be indicated in the “growth” fields. **I)** Finally, whether the plasmid is located within the lab in the form of DNA or e. coli glycerol stocks can also be indicated in the “inventory” fields.

Development • Supplementary information

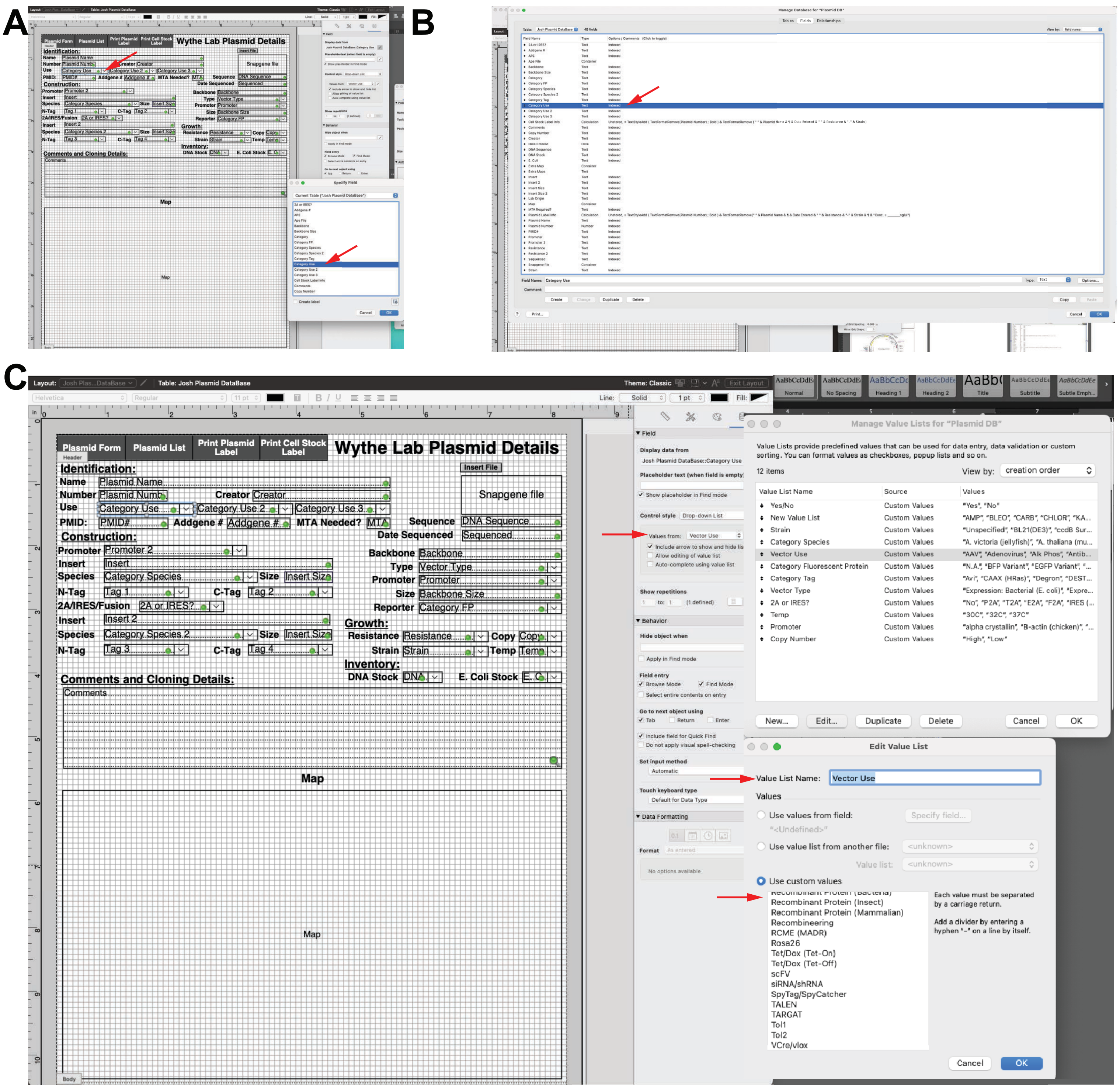

Development • Supplementary information

Fig. S9. Editing the underlying architecture of the FileMaker Database. FileMaker MySQL databases feature and accessible graphical user interface (GUI) that is readily editable and easily manipulated to suit any individual laboratory. **A)** Once the edit database feature is selected, this view will appear. Once can then select a field, in this case “category use” and the related pop-up window will appear on the far right. In this view, select the tower like stacked icon to the far upper right (underlined in blue) and then select the “category use” field below will ensure that the items contained in this list will appear in that field on the database. **B)** Selecting “ok” will ensure that that particular field appears in that box in the normal view of the database. **C)** If you want to actually edit or modify what values appear in that list, then for that field (in this case, “Category Use”, one can see the “Control Style” field near the top that specifies it will appear as a “drop down list”. Below that, the “Values From: Vector Use” lists what table or list the values are actually being pulled from. To see or modify this list, from the pop-up list for “vector use” on the far upper right of the screen, select “edit”. Then the lower pop-up window will appear. In this case, “custom values” are entered by the user, and you can enter whatever use terms are relevant for plasmids within your user group (we suggest listing them in alphabetical order for simple scrolling). Once you have modified the list, select “ok” and then exit out and save the database. Now the “Category Use” dropdown list will contain your novel term(s).

Development • Supplementary information

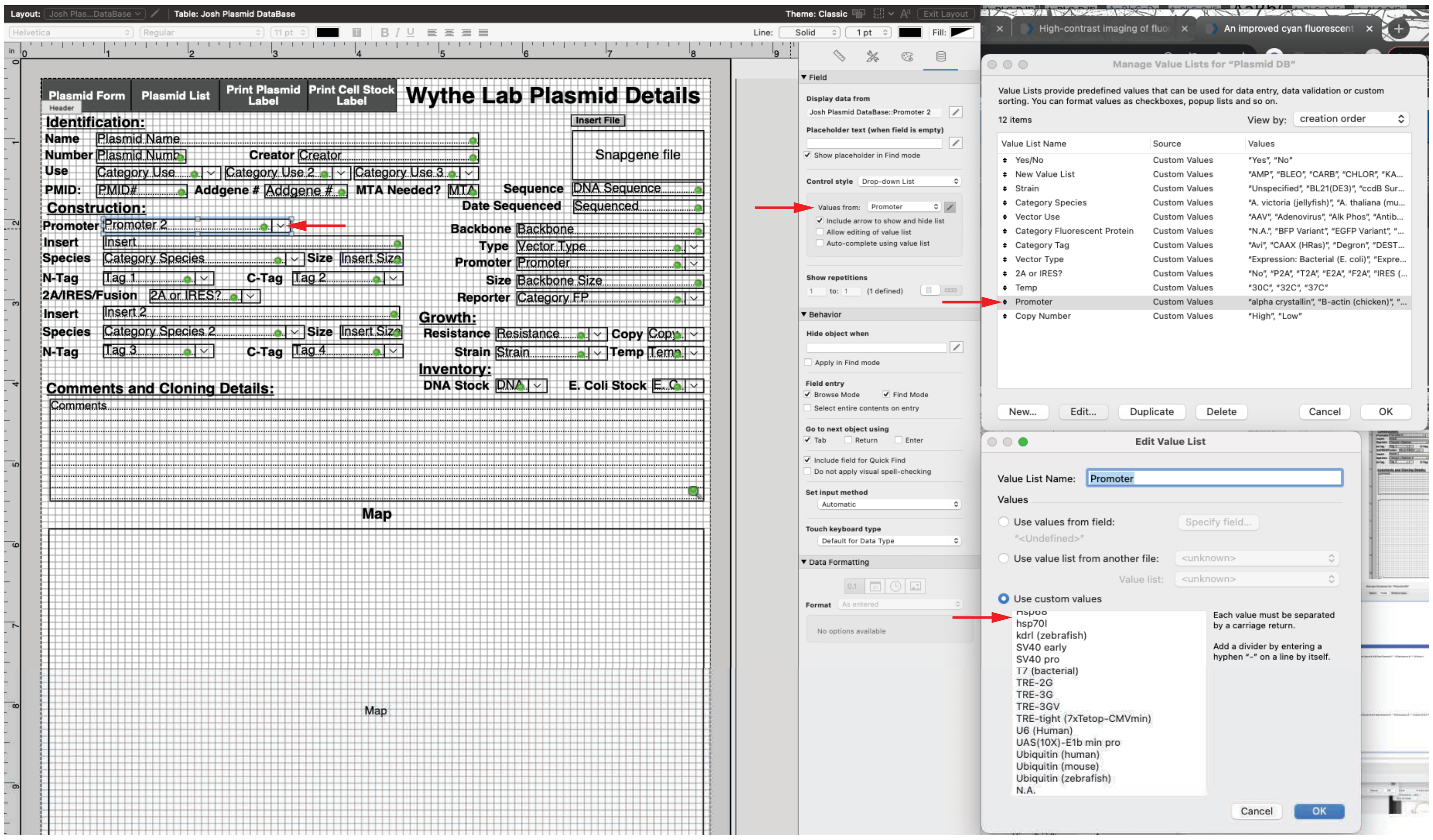

Fig. S10. Modifying the Construction Fields. Similar to the “Category Use” field, the “Construction” fields each feature numerous categories that can be modified (but many of which are already linked to pre-populated dropdown lists). In this case, to modify the list of typically used promoters, in the edit view, select the “Promoter” entry field, then from the pop-up window on the left, using the stacked coins icon, select the “Values From” “Promoter” field. This will bring up the far right pop-up window. On this window, select “promoter” and “edit”, which will bring up the lower right pop-up window. Here, you can add or subtract from the existing list. Once done, simply select “ok” and save and exit from this view to be able to select novel promoters from the dropdown list.

Development • Supplementary information

Aligned using MUSCLE

Consensus

1. (n1)oxStayGold
2. (n2)oxStayGold(c4)_v2
3. mStayGold (J) Miyawaki
4. mBaoJin

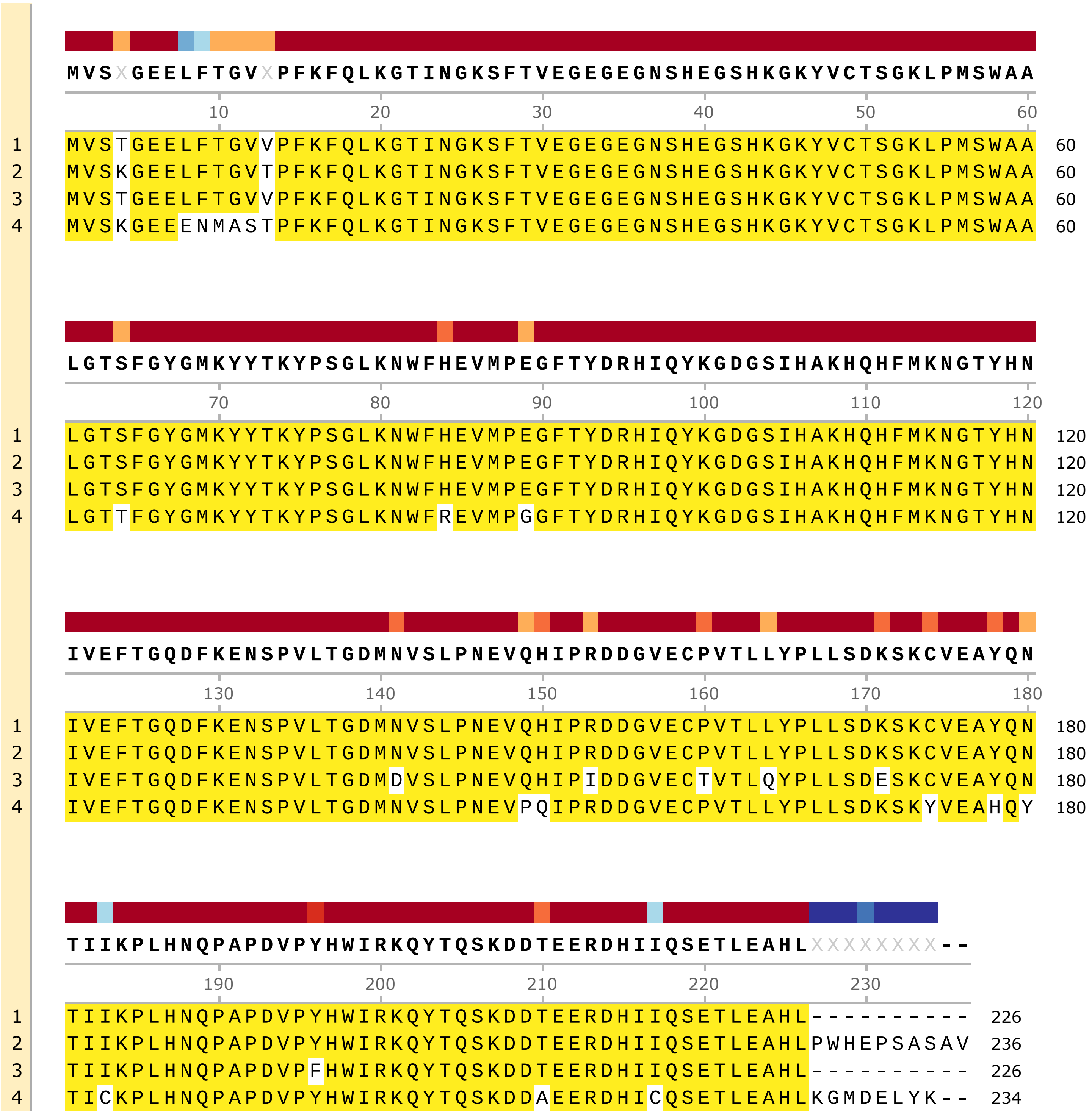

Fig. S11. Comparison of the Amino Acid Composition of StayGold Variants. ClustalW alignment of the amino acid sequence of various StayGold variants. Residues that are conserved are colored yellow.

Development • Supplementary information

Aligned using MUSCLE

Consensus

1. mScarlet
2. mScarlet-I
3. mScarlet-H
4. mScarlet3
5. mScarlet3-H
6. mScarlet3-S2
7. mScarlet-I3

```
  -MDSTEAVIKEFMRFKVHMEGSMNGHEFEIEGEGEGRPYEGTQTAKLKVTKGGPLPFSWD
1 MVSKGEAVIKEFMRFKVHMEGSMNGHEFEIEGEGEGRPYEGTQTAKLKVTKGGPLPFSWD  60
2 MVSKGEAVIKEFMRFKVHMEGSMNGHEFEIEGEGEGRPYEGTQTAKLKVTKGGPLPFSWD  60
3 MVSKGEAVIKEFMRFKVHMEGSMNGHEFEIEGEGEGRPYEGTQTAKLKVTKGGPLPFSWD  60
4 -MDSTEAVIKEFMRFKVHMEGSMNGHEFEIEGEGEGRPYEGTQTAKLRVTKGGPLPFSWD  59
5 -MDSTEAVIKEFMRFKVHMEGSMNGHEFEIEGEGEGRPYEGTQTAKLRVTKGGPLPFSWD  59
6 -MDSTEAVIKEFMRFKVHMEGSMNGHEFEIEGEGEGRPYEGTQTAKLRVTKGGPLPFSWD  59
7 -MDSTEAVIKEFMRFKVHMEGSMNGHEFEIEGEGEGRPYEGTQTAKLKVTKGGPLPFSWD  59

  ILSPQFMYGSRAFTKHPADIPDYWKQSFPEGFKWERVMNFEDGGAVSVTQDTSLEDGTLI
1 ILSPQFMYGSRAFTKHPADIPDYYKQSFPEGFKWERVMNFEDGGAVTVTQDTSLEDGTLI  120
2 ILSPQFMYGSRAFIKHPADIPDYYKQSFPEGFKWERVMNFEDGGAVTVTQDTSLEDGTLI  120
3 ILSPQFMYGSRAFTKHPADIPDYYKQSFPEGFKWERVMNFEDGGAVTVTQDTSLEDGTLI  120
4 ILSPQFMYGSRAFTKHPADIPDYWKQSFPEGFKWERVMNFEDGGAVSVAQDTSLEDGTLI  119
5 ILSPQFMYGSRAFTKHPADIPDYWKQSFPEGFKWERVMNFEDGGAVSVAQDTSLEDGTLI  119
6 ILSPQFQYGSRAFTKHPADIPDYWKQSFPEGFKWERVMNFEDGGAVSVAQDTSLEDGTLI  119
7 ILSPQFMYGSRAFIKHPADIPDYWKQSFPEGFKWERVMIFEDGGTVSVTQDTSLEDGTLI  119

  YKVKLRGTNFPPDGPVMQKKTMGWEASTERLYPEDVVLKGDIKMALRLKDGGRYLADFKT
1 YKVKLRGTNFPPDGPVMQKKTMGWEASTERLYPEDGVLKGDIKMALRLKDGGRYLADFKT  180
2 YKVKLRGTNFPPDGPVMQKKTMGWEASTERLYPEDGVLKGDIKMALRLKDGGRYLADFKT  180
3 YKVKLRGTNFPPDGPVMQKKTMGWEASTERLYPEDGVLKGDIKHALRLKDGGRYLADFKT  180
4 YKVKLRGTNFPPDGPVMQKKTMGWEASTERLYPEDVVLKGDIKMALRLKDGGRYLADFKT  179
5 YKVKLRGTNFPPDGPVMQKKTMGWEASTERLYPEDVVLKGDIKHALRLKDGGRYLADFKT  179
6 YKVKLRGTNFPPDGPVMQKKTMGWEASTERLYPEDVVLKGDIKHALRLKDGGRYLADFKT  179
7 YKVKLRGGNFPPDGPVMQKRTMGWEASTERLYPEDVVLKGDIKMALRLKDGGRYLADFKT  179

  TYKAKKPVQMPGAFNIDRKLDITSHNEDYTVVEQYERSVARHSTGGSGGS--
1 TYKAKKPVQMPGAYNVDRKLDITSHNEDYTVVEQYERSEGRHSTGGMDELYK  232
2 TYKAKKPVQMPGAYNVDRKLDITSHNEDYTVVEQYERSEGRHSTGGMDELYK  232
3 TYKAKKPVQMPGAYNVDRKLDITSHNEDYTVVEQYERSEGRHSTGGMDELYK  232
4 TYRAKKPVQMPGAFNIDRKLDITSHNEDYTVVEQYERSVARHSTGGSGGS--  229
5 TYRAKKPVQMPGAFNIDRKLDITSHNEDYTVVEQYERSVARHSTGGSGGS--  229
6 TYRAKKPVQMPGAFNIDRKLDITSHNEDYTVVEQYERSVARHSTGGSGGS--  229
7 TYKAKKPVQMPGAFNIDRKLDITSHNEDYTVVEQYERSVARHSTGGSGGS--  229
```

Fig. S12. Comparison of the Amino Acid Composition of mScarlet Variants. ClustalW alignment of the amino acid sequence of various mScarlet variants. Residues that are conserved are colored yellow.

Development • Supplementary information

Table S1. Att sites/sequences

Base Sequence	Primer Sequences used for PCR	Uses	Compatible Plasmid
attB4<br>*CAACTTTGTATAGAAAAGTTG*	attB4 (FWD Primer):<br>5’-GGGG ACAACTTTGTATAGAAAAGTTGNN	Creating p5E Entry clones	pDONR P4-P1R
attB1<br>*CAAGTTTGTACAAAAAAGCAGGCT*	attB1r (REV Primer):<br>5’-GGGG ACTGCTTTTTTGTACAAACTTGN	Creating p5E Entry clones	pDONR P4-P1R
attB1<br>*CAAGTTTGTACAAAAAAGCAGGCT*	attB1 (FWD Primer):<br>5'-GGGG ACAAGTTTGTACAAAAAAGCAGGCTNN	Creating pME Entry clones	pDONR221 (attP1-attP2)
attB2<br>*ACCCAGCTTTCTTGTACAAAGTGG*	attB2r (REV Primer):<br>5’-GGGG ACCACTTTGTACAAGAAAGCTGGGTN	Creating pME Entry clones	pDONR221 (attP1-attP2)
attB2<br>*ACCCAGCTTTCTTGTACAAAGTGG*	attB2 FWD Primer:<br>5’- GGGG ACAGCTTTCTTGTACAAAGTGGNN	Creating p3E Entry clones	pDONR P2R-P3
attB3<br>*CAACTTTGTATAATAAAGTTG*	attB3r REV Primer:<br>5’- GGGG ACAACTTTGTATAATAAAGTTGN	Creating p3E Entry clones	pDONR P2R-P3

Development • Supplementary information

Table S2. Novel p5E vectors

Plasmid ID #	Name	Description	Uses	Figure	Origin Species	Lab Origin / Citation
JDW 912	p5E-CAGGS	CAGGS promoter	Strong, Ubiquitous Mammalian Expression	Figure 2	Synthetic: CMV, Chicken, rabbit	Kwan Lab / Gillespie et al 2025
JDW 1164	p5E-EF1a	Human EF-1a core promoter and intron 1	Moderate Mammalian Expression	Figure 2	Homo sapiens	Wythe Lab / Gillespie et al 2025
JDW 1319	p5E-EFS (EF1a core)	Human core EF-1a promoter	Moderate to low mammalian expression		Homo sapiens	Wythe Lab / Gillespie et al 2025
JDW 1461	p5E-hs_UbiC-pro	Human Ubiquitin promoter	Moderate Mammalian Expression		Homo sapiens	Wythe Lab / Gillespie et al 2025
JDW 1087	p5E-TetO$_{8x}$-CMVmin	8 tet operons and a minimal CMV promoter	Dox-inducible promoter		Synthetic: tet operon, CMV	Wythe Lab / Gillespie et al 2025
JDW 1163	p5E-TRE-3GV	3rd generation Tet-On promoter	Dox inducible promoter		Synthetic: tet operon, pTight, CMV	Wythe Lab / Gillespie et al 2025
JDW 1241	p5E 5X C120 c-fos min pro	5 TAEL TF binding sites and a minimal c-Fos promoter	TAEL responsive promoter for use in zebrafish		Synthetic: C120, mus musculus	Wythe Lab / Gillespie et al 2025
JDW 1254	p5E lexOP-c-fos min pro	Lex Operon upstream of c-fos minimal promoter	RU486-inducible promoter	Figure 4	Synthetic: lex Operon, mus musculus	Wythe Lab / Gillespie et al 2025
JDW 1239	p5E-mmCdh5pro	Murine Cdh5 promoter	Pan endothelial promoter		Mus musculus	Wythe Lab / Gillespie et al 2025
JDW 1046	p5E-hs_ICAM_full	Human ICAM2 promoter	Pan endothelial promoter		Homo sapiens	Wythe Lab / Gillespie et al 2025
JDW 1047	p5E-hs_ICAM_short	Human minimal ICAM2 promoter	Compact, pan endothelial promoter		Homo sapiens	Wythe Lab / Gillespie et al 2025
JDW 1120	p5E-hs_Cldn5	Human Claudin 5 promoter	Pan endothelial promoter		Homo sapiens	Wythe Lab / Gillespie et al 2025
JDW 1366	p5E-mm_Dll4-F2-E1b-b-globin intron	Murine Dll4 F2 arterial enahncer and E1b minimal promoter and b-globin intron	Arterial-specific promoter for use in zebrafish		Mus musculus	Wythe Lab / Gillespie et al 2025

Development • Supplementary information

JDW 1148	p5E-dr_myl7_pro	Zebrafish myl7 / cmlc2 promoter and exon 1	Pan cardiomyocyte promoter for use in zebrafish		Danio rerio	Wythe Lab / Gillespie et al 2025
JDW 1365	p5E-dr_Unc	Zebrafish *unc-503* promoter with a minimal c-fos promoter and b-globin intron	Pan muscle promoter for use in zebrafish		Danio rerio	Wythe Lab / Gillespie et al 2025
JDW 1208	p5E-hs_TNNT2	Human TNNT2 promoter	Pan cardiomyocyte promoter for mammalian studies		Homo sapiens	Wythe Lab / Gillespie et al 2025
JDW 1182	p5E-hs_GLAST	Human GLAST/EEAT1 promoter	Radial glia progenitors, astrocytes	Figure 8	Homo sapiens	Wythe Lab / Gillespie et al 2025
JDW 1237	p5E-MCS-c-fos-b-globin	Multiple cloning site followed by murine c-fos minimal promoter and b-globin intron	Minimal promoter for enhancer validation in zebrafish		Synthetic: mus musculus, rabbit	Wythe Lab / Gillespie et al 2025
JDW 1320	p5E-Crestin	Zebrafish neural crest enhancer element upstream of c-fos minimal promoter and globin intron	Neural crest enhancer / promoter for use in zebrafish		Danio rerio	Wythe Lab / Gillespie et al 2025

Development • Supplementary information

Table S3. Novel pME vectors

Plasmid ID #	Name	Description	Uses	Figure	Species	Lab Origin / Citation
JDW 455	pME-MCS (WPD)	Novel MCS with EcoRI-SalI-BamHI-KpnI-SmaI-NotI-XhoI-EcoRI	Subcloning for new pME / middle entry clones	NA	NA	Devine Lab / Gillespie et al 2025
JDW 830	pME-V5-mTagBFP2	V5 tagged mTagBFP2	Cytosolic blue fluorescent reporter		Synthetic; Entacmaea quadricolor	Wythe Lab / Gillespie et al 2025
JDW 1151	pME-AmCyan	AmCyan	Cytosolic blue fluorescent reporter		Synthetic; Anemonia majano	Wythe Lab / Gillespie et al 2025
JDW 669	pME-mRuby2-3xMyc-STOP	mRuby2 with 3x MYC tag	Cytosolic red fluorescent reporter		Synthetic; Entacmaea quadricolor	Wythe Lab / Gillespie et al 2025
JDW 1323	pME-V5-mScarlet-I	V5 tagged mScarlet-I	Cytosolic far red fluorescent reporter		Synthetic	Wythe Lab / Gillespie et al 2025
JDW 484	pME nls BFP Flag WPRE bGH pA	3xNLS mTagBFP with c-terminal FLAG tag	Nuclear blue fluorescent reporter	Figure S3	Synthetic; Entacmaea quadricolor	Wythe Lab / Gillespie et al 2025
JDW 488	pME-nls-EGFP-WPRE-bGH-pA	3xNLS-EGFP	Nuclear green fluorescent reporter		Synthetic; Aequorea victoria	Wythe Lab / Gillespie et al 2025
JDW 485	pME-nls-mKate2-V5-WPRE-bGH-pA	3xNLS-mKate2 with c-terminal V5 tag	Nuclear red fluorescent reporter		Synthetic; Entacmaea quadricolor	Wythe Lab / Gillespie et al 2025
JDW 1150	pME-H2B-mCerulean	Histone H2B fused to mCerulean	Stable nuclear localized Cerulean fluorescent reporter		Synthetic; Aequorea victoria	Wythe Lab / Gillespie et al 2025
JDW 1383	pME-H2B-mBaoJin-WPRE	Monomeric green fluorescent reporter	Cytosolic green fluorescent reporter		Synthetic; Cytaeis uchidae	Wythe Lab / Gillespie et al 2025
JDW 1354	pME-H2B-V5-n2StayGoldC4	Histone H2B fused to V5 tagged (n2)StayGold(C4)	Nuclear StayGold fluorescent reporter		Synthetic; Cytaeis uchidae	Wythe Lab / Gillespie et al 2025
JDW 1384	pME-H2B-mStayGold-WPRE	Histone H2B fuseed to monomeric StayGold variant	Nuclear monomeric StayGold fluorescent reporter		Synthetic; Cytaeis uchidae	Wythe Lab / Gillespie et al 2025
JDW 1513	pME-H2B-mScarlet-3H	Histone H2B fuseed to monomeric	Nuclear monomeric mScarlet3 fluorescent reporter	Figure S3	synthetic, mammalian codon optimized	Wythe Lab / Gillespie et al 2025

Development • Supplementary information

		mScarlet-3 variant M163H				
JDW 1514	pME-H2B-mScarlet-3S2 (mScarlet3-S2)	Histone H2B fuseed to monomeric mScarlet-3 variant M63Q, M163H	Nuclear monomeric mScarlet3 fluorescent reporter		synthetic, mammalian codon optimized	Wythe Lab / Gillespie et al 2025
JDW 1418	pME-H2B-mCardinal	Histone H2B fused to mCardinal	Nuclear localized far red reporter		Synthetic; Entacmaea quadricolor	Wythe Lab / Gillespie et al 2025
JDW 926	pME-Luciferase-H2A-mCherry	Dual nuclear red and cytosolic luciferase reporter	Luciferase bioilluminescence and fluorescent reporter	Figure 5, Figure 7	H2A.Z=danio rerio Luciferase = Firefly mCherry = Synthetic; Discosoma sp.	Wythe Lab / Gillespie et al 2025
JDW 681	pME-Sun1-2xsfGFP-6xMyc-pA	Sun1 fusion to 2 copies of sfGFP with 6 c-terminal Myc tags	Nuclear envelope green fluorescent reporter and affinity tag	Figure 1	Synthetic; Aequorea victoria	Wythe Lab / Gillespie et al 2025
JDW 1312	pME LaminB Vhh mNeonGreen-HA-pA	Lamin B nanobody fused to mNeonGreen	Visualizing nuclear lamina with green fluorescent protein		Lamin B=homo sapiens mNeonGreen= Synthetic; Branchiostoma lanceolatum	Wythe Lab / Gillespie et al 2025
JDW 1007	pME-Golgi-BFP_P2A_H2A-iRFP	GalT-mTagBFP tagged with HA followed by H2A iRFP	Blue golgi reporter and infared iRFP nuclear reporter		H2A.Z=danio rerio; GalT=homo sapiens	Wythe Lab / Gillespie et al 2025
JDW 1079	pME-Golgi-BFP_P2A_H2A-tdiRFP	GalT-mTagBFP tagged with HA followed by H2A tdiRFP	Blue golgi reporter and infared tdiRFP nuclear reporter	Figure 1	H2A.Z=danio rerio; GalT=homo sapiens	Wythe Lab / Gillespie et al 2025
JDW 1183	pME-myr-mTagBFP2-Flag	Myristolated mTagBFP2 with a c-terminal Flag tag	Cell membrane blue fluorescent reporter		Synthetic; Entacmaea quadricolor	Wythe Lab / Gillespie et al 2025
JDW 678	pME P2A Lifeact mRuby2-3xMyc	MCS followed by P2A and Lifeact mRuby2-3xMyc	For making bicistronic mRuby2 F-actin reporters		Lifeact = Saccharomyces cerevisiae mRuby2 = Synthetic; Entacmaea quadricolor	Wythe Lab / Gillespie et al 2025
JDW 1246	pME Lifeact mRuby2-3xMyc	Lifeact mRuby2 with 3, c-terminal Myc tags	Red fluorescent F-actin reporter	Figure S3	Lifeact = Saccharomyces cerevisiae mRuby2 = Synthetic; Entacmaea	Wythe Lab / Gillespie et al 2025

Development • Supplementary information

					quadricolor	
JDW 1247	pME Lifeact-mScarlet-I-3xHA	Lifeact mScarlet-I tagged with HA	Red fluorescent F-actin reporter		Lifeact = Saccharomyces cerevisiae<br>mScarlet-I = Synthetic	Wythe Lab / Gillespie et al 2025
JDW 1322	pME-Lifeact-EGFP-3xHA	Lifeact EGFP tagged with HA	Green fluorescent F-actin reporter		Lifeact = Saccharomyces cerevisiae<br>EGFP = Synthetic; Aequorea victoria	Wythe Lab / Gillespie et al 2025
JDW 1215	pME-Actin-Vhh-sfGFP	Actin nanobody fused to sfGFP reporter	Green fluorescent actin reporter		Actin-Vhh = Vicugna pacos<br>sfGFP = Synthetic; Aequorea victoria	Wythe Lab / Gillespie et al 2025
JDW 1216	pME-Actin-Vhh-sfGFP-P2A-HA-iRFPcaax	Actin nanobody fused to sfGFP followed by iRFPcaax	Green fluorescent actin reporter and infared cell membrane reporter		Actin-Vhh = Vicugna pacos<br>sfGFP = Synthetic; Aequorea victoria<br>iRFP = Synthetic; Rhodopseudomonas palustris	Wythe Lab / Gillespie et al 2025
JDW 1311	pME-Actin-Vhh-sfGFP-P2A-HA-tdiRFPcaax	Actin nanobody fused to sfGFP followed by tdiRFPcaax	Green fluorescent actin reporter and tandem dimer infared cell membrane reporter		Actin-Vhh = Vicugna pacos<br>sfGFP = Synthetic; Aequorea victoria<br>iRFP = Synthetic; Rhodopseudomonas palustris	Wythe Lab / Gillespie et al 2025
JDW 1223	pME Actin Vhh Halo Tag	Actin Nanobody fused to a flexible linker and Halo Tag	Visualizing actin and use with Halo dyes	Figure 1	Actin-Vhh = Vicugna pacos	Wythe Lab / Gillespie et al 2025
JDW 1240	pME-Actin-Vhh-mCherry	Actin nanobody fused to mCherry	Visualizing actin with red fluorescent reporter		Actin-Vhh = Vicugna pacos<br>mCherry = Synthetic; Discosoma sp.	Wythe Lab / Gillespie et al 2025
JDW 1248	pME Actin Vhh mNeonGreen-HA-pA	Actin nanobody fused to mNeonGreen	Visualizing actin with green fluorescent reporter	Figure 1, Figure S1	Actin-Vhh = Vicugna pacos<br>mNeonGreen = Synthetic; Branchiostoma lanceolatum	Wythe Lab / Gillespie et al 2025
JDW 1356	pME-mScarlet3-Giantin	V5 tagged mScarlet3 fused to	For visualizing the Golgi with red		mScarlet3 = Synthetic	Wythe Lab / Gillespie

Development • Supplementary information

		Giantin	fluorescent protein			et al 2025
JDW 1355	pME-StayGold(c4)-Giantin	StayGold(c4) fused to V5 tagged Giantin	For visualizing the Golgi with green fluorescent protein		StayGold(c4) = synthetic	Wythe Lab / Gillespie et al 2025
JDW 1508	pME-mCerulean3-TUBB1A	mCerulean3 fused to TUBB1A	For visualizing Tubulin in cells		mCerulean3 = Synthetic; Aequorea victoria TUBB1A = homo sapiens	Wythe Lab / Gillespie et al 2025
JDW 1488	pME-FastFUCCI	mKO2-Cdt1(30-120) T2A mAzamiGreen-Gem(1-110)	For visualizing cell cycle state in mammalian cells	Figure 2, Figure S3	Cdt1, Geminin = homo sapiens	Wythe Lab / Gillespie et al 2025
JDW 1399	pME-FastFucci-P2A-H2A-tdiRFP	Mammalian FastFUCCI reporter followed by H2A tdIRFP	For visualizing cell cycle state and nuclear tandem dimer infared reporter		Cdt1, Geminin = homo sapiens; H2A.Z=danio rerio	Wythe Lab / Gillespie et al 2025
JDW 1464	pME-zFUCCI	mCerulean-zGem(1-100) P2A mCherry-zCdt1(1-190)	For visualizing cell cycle state in zebrafish	Figure 2	Cdt1, Geminin = danio rerio	Wythe Lab / Gillespie et al 2025
JDW 1380	pME-zFUCCI-P2A-H2A-tdiRFP	Zebrafish FUCCI reporter followed by H2A tdIRFP	For visualizing cell cycle state and nuclear tandem dimer infared reporter		Cdt1, Geminin, H2A.Z = danio rerio	Wythe Lab / Gillespie et al 2025
JDW 937	pME-LexA-mODC (no LexOp)	Destabilized LexA	Destabilized Lex transactivator	Figure 4	LexA = e. coli	Wythe Lab / Gillespie et al 2025
JDW 1348	pME-LexA-mODC-LexOp-35SCaMV	Destablized LexA and LexOperon with minimal CaMV 35S promoter	All in one cassette for RU486 driven gene expression	Figure 4	Synthetic: cauliflower, lex Operon	Wythe Lab / Gillespie et al 2025
JDW 1359	pME-LexA-mODC-LexOP-c-Fos	Destablized LexA and LexOperon with 3' minimal murine c-Fos promoter	All in one cassette for RU486 driven gene expression	Figure 4	Synthetic: lex Opern, mus musculus	Wythe Lab / Gillespie et al 2025
JDW 1109	pME TAEL2.0-5xC120-minpro	TAEL2.0 transcription factor, then SV40 pA, pause, NOS terminator, 5xC120 response element,	All in one binary TAEL and effector domain clone	Figure 3	Synthetic: C120, agrobacterium, mus musculus	Wythe Lab / Gillespie et al 2025

Development • Supplementary information

		minimal murine c-Fos promoter				
JDW 1324	pME-TAEL2.0	TAEL2.0 transcription factor, SV40 pA, pause, NOS terminator	For making binary TAEL system vectors, this is for generating a tissue-specific driver.	Figure 3	Synthetic	Wythe Lab / Gillespie et al 2025
JDW 1119	pME-LiCre	Light inducible Cre	Cre/lox	Figure 3	Synthetic	Wythe Lab / Gillespie et al 2025
JDW 1149	pME-3xNLS-$(G_2SG_2)_2$-Cre	3xNLS-MCS-Gly-Gly-Ser-Gly-Gly-linker-Cre recombinase	Cre/lox		Cre = bacteriophage	Wythe Lab / Gillespie et al 2025
JDW 1203	pME iCre-I	loxP flanked, self inactivating Cre that contains an intron to prevent recombination in bacteria	Cre/lox		Cre = bacteriophage	Wythe Lab / Gillespie et al 2025
JDW 918	pME-3xNLS-mTagBFP2-Cre	3xNLS-mTagBFP2-Cre fusion	Cre/lox		Cre = bacteriophage BFP = Synthetic; Anemonia majano	Wythe Lab / Gillespie et al 2025
JDW 1329	pME nls EGFP-Cre	NLS-EGFP-Cre fusion	Cre/lox		Cre = bacteriophage EGFP = Synthetic; Aequorea victoria	Wythe Lab / Gillespie et al 2025
JDW 920	pME-mTurquoise-P2A-iCre	mTurquoise P2A iCre (codon optimized)	Cre/lox		Cre = bacteriophage (codon optimized) mTurquoise = Synthetic; Aequorea victoria	Wythe Lab / Gillespie et al 2025
JDW 921	pME-EGFP-P2A-iCre	EGFP P2A iCre (codon optimized)	Cre/lox		Cre = bacteriophage (codon optimized) EGFP = Synthetic; Aequorea victoria	Wythe Lab / Gillespie et al 2025
JDW 917	pME-mScarlet-I-P2A-iCre	mScarlet-I P2A-iCre (codon optimized)	Cre/lox		Cre = bacteriophage (codon optimized) EGFP = Synthetic	Wythe Lab / Gillespie et al 2025
JDW 869	pME-lox-3xSTOP-lox	loxP flanked SV40 polyA, bGH polyA, and SV40 pA stop cassette	Cre/lox		Synthetic	Wythe Lab / Gillespie et al 2025

Development • Supplementary information

JDW 1232	pME-lox-3xNLS-mCherry-V5-SV40pA-stop-lox	loxP flanked 3xNLS-mKate-V5-2xSTOP cassette	Cre/lox		Synthetic	Wythe Lab / Gillespie et al 2025
JDW 31	pME-DRE-nls	Dre recombinase with c-terminal 1x NLS	Dre / rox	Figure 6	D6 Phage (codon optimized)	Wythe Lab / Gillespie et al 2025
JDW 1344	pME-DreERT2	Dre recombinase fused to mutant ERT2 for tamoxifen-inducible recombinase activity	Dre / rox		Synthetic; D6 Phage (codon optimized)	Wythe Lab / Gillespie et al 2025
JDW 1167	pME-rox-3xNLS-mKate-V5-2xSTOP-rox	Rox flanked 3xNLS-mKate-V5-2xSTOP cassette	Dre / rox	Figure 6	Synthetic mKate = synthetic; Entacmaea quadricolor	Wythe Lab / Gillespie et al 2025
JDW 1098	pME-3x-FLAG-Ubiquitin	3x FLAG tagged human Ubiquitin	Monitor Ubiquitylation status		Ubiquitin=homo sapiens	Wythe Lab / Gillespie et al 2025
JDW 1210	pME-myc-BirA	Myc tagged BirA (R118G)	For BioID proximity labelling		e. coli (codon optimized)	Wythe Lab / Gillespie et al 2025
JDW 1095	pME-Myc-BioID2-MCS1	Myc-tagged BIOID2 construct	For BioID proximity labelling		Aquifex aeolicus	Wythe Lab / Gillespie et al 2025
JDW 1298	pME-Myc-BioID2-MCS2	Myc-tagged BIOID2 construct	For BioID proximity labelling		Aquifex aeolicus	Wythe Lab / Gillespie et al 2025
JDW 889	pME-3xHA-BirA-T2A-mCherry-caax	Biotin Ligase followed by T2A and mCherry membrane reporter	Biotinylation and fluorescent cell membrane reporter		BirA = e. coli (codon optimized) mCherry = synthetic; Discosoma sp.	Wythe Lab / Gillespie et al 2025
JDW 1230	pME EFS-rtTA/rtTA3	rtTA-3rd generation transactivator	Tet-On / Dox	Figure 5	Synthetic	Wythe Lab / Gillespie et al 2025
JDW 679	pME-hyPBase-WPRE	Hyperactive PBase	PBase / *piggyBac*		Synthetic; Trichoplusia ni	Wythe Lab / Gillespie et al 2025
JDW 1231	pME-EGFP-miR-MCS	Intron containg EGFP with MCS for inserting microRNAs	microRNA expression vector		Synthetic; Aequorea victoria	Wythe Lab / Gillespie et al 2025
JDW 8	pME-EGFP-mm-miR-500	EGFP with an intron containing murine miR-500	microRNA expression vector		mIR-500=mus musculus	Wythe Lab / Gillespie et al 2025

Development • Supplementary information

JDW 1401	pME-EGFP-hs-miR-126	EGFP with an intron containing human miR-126	microRNA expression vector		miR-126=homo sapiens	Wythe Lab / Gillespie et al 2025
JDW 419	pME-B-catenin-S33Y-FLAG	Dominant active B-Catenin with a c-terminal flag tag	Activate canonical Wnt signaling		homo sapiens	Wythe Lab / Gillespie et al 2025
JDW 812	pME-V5-mClover-KRAS4A-WT	mClover fused to human KRAS4A	Over expression of green FP fused KRAS		homo sapiens	Wythe Lab / Gillespie et al 2025
JDW 813	pME-V5-mClover-KRAS4A-G12D	mClover fused to human mutant KRAS4A-G12D	Over expression of green FP fused mutant KRAS		homo sapiens	Wythe Lab / Gillespie et al 2025
JDW 822	pME-V5-mScarlet-I-KRAS4A-WT	mScarlet fused to human KRAS4A	Over expression of red FP fused KRAS		homo sapiens	Wythe Lab / Gillespie et al 2025
JDW 823	pME-V5-mScarlet-I-KRAS4A-G12V	mScarlet fused to human KRAS4A G12V	Over expression of red FP fused mutant KRAS		homo sapiens	Wythe Lab / Gillespie et al 2025
JDW 830	pME-V5-mTagBFP2	mTagBFP2	Overexpression of V5 tagged mTagBFP2		synthetic, mammalian codon optimized	Wythe Lab / Gillespie et al 2025
JDW 831	pME-V5-mTagBFP2-KRAS4A-WT	mTagBFP2 fused to human KRAS4A	Over expression of blue FP fused KRAS		homo sapiens	Wythe Lab / Gillespie et al 2025
JDW 832	pME-V5-mTagBFP2-KRAS4A-G12D	mTagBFP2 fused to human KRAS4A-G12D	Over expression of blue FP fused mutant KRAS		homo sapiens	Wythe Lab / Gillespie et al 2025
JDW 1441	pME-EGFP-MYC	EGFP fused to human c-MYC	Overexpression of green FP fused to MYC		homo sapiens	Wythe Lab / Gillespie et al 2025

Development • Supplementary information

Table S4. Novel p3E vectors

Plasmid ID #	Name	Description	Uses	Figure	Origin Species	Lab Origin / Citation
JDW 922	p3E-WPRE-SV40-pA	Woodchuck Herpes simplex virus regulatory element (WPRE) upstream of SV40 pA	Stabilize mRNA, poly(A) signal		viral	Wythe Lab / Gillespie et al 2025
JDW 1221	p3E-WPRE-bGH-pA	Woodchuck Herpes simplex virus regulatory element (WPRE) upstream of Bovine growth hormone polyA	Stabilize mRNA, poly(A) signal		viral	Wythe Lab / Gillespie et al 2025
JDW 1222	p3E-EF1a-pA	Rat EF-1a polyA	poly(A) signal		human	Wythe Lab / Gillespie et al 2025
JDW 1417	p3E-mCherry-SV40-pA	mCherry followed by SV40 polyA	mCherry reporter		Synthetic; Discosoma sp	
JDW 967	p3E-H2A_mCherry_SV40pA	H2A mCherry fusion followed by SV40 late polyA	Nuclear red fluorescent reporter		Synthetic; Discosoma sp	Wythe Lab / Gillespie et al 2025
JDW 968	p3E-V5-mScarlet-I_SV40pA	V5 tagged mScarlet-I followed by polyA	Nuclear far red fluorescent reporter		Synthetic	Wythe Lab / Gillespie et al 2025
JDW 1318	p3E-V5-mClover3-pA	V5 tagged mClover3 followed by an SV40-pA	Cytosolic green fluorescent reporter		Synthetic; Aequorea victoria	Wythe Lab / Gillespie et al 2025
JDW 871	p3E-V5-mTagBFP2	V5 tagged mTagBFP2	Cytosolic blue fluorescent reporter		Synthetic; Entacmaea quadricolor	Wythe Lab / Gillespie et al 2025
JDW 1214	p3E bGH-pA-EFS-rtTA/rtTA3	bovine growth hormone polyA followed by a human EFS promoter driving rtTA-3rd generation transactivator	Tet-On / Dox		synthetic	Wythe Lab / Gillespie et al 2025
JDW 1188	p3E mCherry-T2A-hsHRAS-G12V-pA	mCherry T2A followed by human HRAS G12V	Cytosolic red fluorescent reporter and constitutively active HRAS	Figure 8	mCherry = Synthetic; Discosoma sp HRAS = homo sapiens	Wythe Lab / Gillespie et al 2025

Development • Supplementary information

JDW 1225	p3E-Actin-Vhh-mNeonGreen-HA	Actin nanobody fused to mNeonGreen with a c-terminal HA tag	Green fluorescent protein labelling of actin	Figure 6	Actin-Vhh = Vicugna pacos mNeonGreen = Synthetic; Branchiostoma lanceolatum	Wythe Lab / Gillespie et al 2025
JDW 1256	p3E-IRES-H2A-mCherry-SV40pA	IRES-H2A-mCherry	IRES stable nuclear red fluorescent reporter		H2A.Z = danio rerio mCherry = Synthetic; Discosoma sp.	Wythe Lab / Gillespie et al 2025
JDW 1360	p3E-IRES-3xNLS-mScarlet-I3-pA	IRES-FLAG- NLS-mScarlet-I3	IRES nuclear red fluorescent reporter		Synthetic	Wythe Lab / Gillespie et al 2025
JDW 1361	p3E-IRES-3xNLS-mTagBFP2-pA	IRES-FLAG-NLS-mTagBFP2-pA	IRES nuclear blue fluorescent reporter	Figure 2	Synthetic; Entacmaea quadricolor	Wythe Lab / Gillespie et al 2025
JDW 1364	p3E-IRES-(n2)StayGold(c4)-pA	IRES V5-tagged StayGold	IRES V5 tagged stable green fluorescent reporter		Synthetic; Cytaeis uchidae	Wythe Lab / Gillespie et al 2025
JDW 1509	p3E-IRES-Luciferase-WPRE-bGH-pA	IRES Luciferase reporter	bioluminescent reporter firefly luciferase (luc2 / luc+)		synthetic luc2 version of the luciferase gene	Wythe Lab / Gillespie et al 2025

Development • Supplementary information

Table S5. Novel Destination vectors

Plasmid ID #	Name	Description	Uses	Figure	Origin Species	Lab Origin / Citation
JDW 471	pCAGEN-DEST	CAGGS promoter driven DEST vector for pME inserts	Strong expression in mammalian cells	Figure 5, Figure S3	Synthetic; CMV (viral), b-actin (chicken), b-globin (rabbit)	Devine Lab / Gillespie et al 2025
JDW 1309	pCAGEN-WPRE-DEST	pCAGEN-DEST with a WPRE upstream of the bGH polyA for enhanced mRNA stability	Strong mammalian expression	Figure 5	Synthetic; CMV (viral), b-actin (chicken), b-globin (rabbit)	Wythe Lab / Gillespie et al 2025
JDW 491	pCAGEN-DEST-IRES-myr-BFP	CAGGS promoter driven DEST vector with 3' IRES myristoylated mTagBFP reporter	Strong mammalian expression with bicistronic membrane BFP reporter	Figure 2	CAGGS = Synthetic; CMV (viral), b-actin (chicken), b-globin (rabbit) BFP = Synthetic; Entacmaea quadricolor	Wythe Lab / Gillespie et al 2025
JDW 495	pCAGEN-DV-IRES-myr-EGFP	CAGGS promoter driven DEST vector with 3' IRES myristoylated EGFP reporter	Strong mammalian expression with bicistronic membrane GFP reporter	Figure S3	CAGGS = Synthetic; CMV (viral), b-actin (chicken), b-globin (rabbit) EGFP = Synthetic; Aequorea victoria	Wythe Lab / Gillespie et al 2025
JDW 494	pCAGEN-DV-IRES-myr-mKate	CAGGS promoter driven DEST vector with 3' IRES myristoylated mKate2 reporter	Strong mammalian expression with bicistronic membrane RFP reporter		CAGGS = Synthetic; CMV (viral), b-actin (chicken), b-globin (rabbit) mKate = Synthetic; Entacmaea quadricolor	Wythe Lab / Gillespie et al 2025
JDW 472	pCAGGS-Rox-3xNLS-mKate-V5-stop-Rox-DEST-NEO	CAGGS promoter driving rox flanked RFP "switch" Destination Dre reporter vector (pCAGGS-rsr-nK-	Dre / rox fluorescent reporter	Figure 6	CAGGS = Synthetic; CMV (viral), b-actin (chicken), b-globin (rabbit) mKate =	Wythe Lab / Gillespie et al 2025 PMID: 25296024

Development • Supplementary information

		DEST-FRT-PGK-NEO-FRT)			Synthetic; Entacmaea quadricolor	
JDW 26	pβ-MHC-promoter DV	Murine b MHC promoter for embryonic and postnatal cardiomyocyte expression compatible with pME inserts.	For cardiomyocyte expression		mus musculus	Wythe Lab / Gillespie et al 2025
JDW 7	pXMLC2-Dest	Xenopous myosin light chain 2 promoter for embryonic and adult cardiomyocyte expression compatible with pME inserts.	For cardiomyocyte expression		Xenopous	Wythe Lab / Gillespie et al 2025
JDW 476	pMef2c-AHF-DV	MEF2c anterior heart field F6 enhancer fragment compatible with pME inserts.	For anterior second heart field expression		mus musculus	Wythe Lab / Gillespie et al 2025 PMID: 25296024
JDW 1009	pGLAST-DEST	Glast (Slc1a3) promoter driven Dest vector compatible with pME inserts.	For expression in radial glial and their descendants	Figure 8	homo sapien	Wythe Lab / Gillespie et al 2025
JDW 1205	pB-DEST	*piggyBac* ITR flanked multisite Gateway compatible Dest vector for recombination, for p5E-pME-p3E insertion	3 insert, multisite Gateway compatible destination vector for stable integration in cells using *piggybac* transposase.	Figure 8	Synthetic	Wythe Lab / Gillespie et al 2025

Development • Supplementary information

JDW 1206	pB-2xIns-DEST	*piggyBac* ITR flanked multisite Gateway compatible Dest vector for recombination, with 2x flanking cHS4 insulators, for p5E-pME-p3E insertion	3 insert, multisite Gateway compatible destination vector for stable integration in cells using *piggyBac* transposase with insulators to prevent transgene silencing.		Synthetic	Wythe Lab / Gillespie et al 2025
JDW 925	pB-Puro-DEST	*piggyBac* ITR flanked multisite Gateway compatible Dest vector for recombation with p5E-pME-p3E with PURO resistance cassette	Multisite Gateway assembly and stable genomic integration, Puromycin selection for making stable lines		Synthetic	Wythe Lab / Gillespie et al 2025
JDW 940	pB-Neo-DEST	*piggyBac* ITR flanked multisite Gateway compatible Dest vector for recombation with p5E-pME-p3E with NEO resistance cassette	Multisite Gateway assembly and stable genomic integration, Neomycin selection for making stable lines		Synthetic	Wythe Lab / Gillespie et al 2025
JDW 1130	pB Tet-On 2.0 (pB-TetOn-DEST-hEF1a-mODC-rtTA-IRES-NEO)	TetOn-DEST vector with full length hEF1a promoter driving destabilized rtTA IRES-NEO	*piggyBac* Destination vector for Tet-On expression	Figure 7	EF1a = homo sapien	Wythe Lab / Gillespie et al 2025
JDW 931	pB Tet-On 3.0 (pB-TetOn-DEST-EFS-mODC-rtTA-IRES-NEO)	TetOn-DEST vector with EFS promoter driving destabilized rtTA IRES-NEO	*piggyBac* Destination vector for Tet-On expression	Figure 7	EFS = homo sapien	Wythe Lab / Gillespie et al 2025

Development • Supplementary information

JDW 936	pB Tet-Off (pB-TRE-FLEX-DEST-EFS-d2tTA)	Cre-dependent, TetOff-DEST vector with downstream WPRE, EFS driving destabilized tTA	*piggyBac* Destination vector for Cre-dependent, Tet Off expression.	Figure 7	EFS = homo sapien	Wythe Lab / Gillespie et al 2025
JDW 1002	pDestTol2-Hsp70-zCreI-TagBFP2-d2-2xins	hsp70 promoter drives codon optimized zCre with an internal intron fused to BFP with mODC tag to promote protein turnover, insulators separate hsp70 from upstream elements	Multisite Tol2 Cloning, zebrafish transgenesis		Danio rerio	Wythe Lab / Gillespie et al 2025
JDW 1184	pDestTol2-Hsp70-zCreI-mTagBFP2-2xIns-	Removed the mODC tag from zCreI-mTagBFP2	Multisite Tol2 Cloning, zebrafish transgenesis	Figure 8	Danio rerio	Wythe Lab / Gillespie et al 2025
JDW 1218	pDestTol2-fli1ep-zCreI-BFP	fli1ep drives codon optimized zCre with an internal intron fused to BFP in the endothelium, insulators separate fli1ep from upstream elements	Multisite Tol2 Cloning, zebrafish transgenesis		Danio rerio	Wythe Lab / Gillespie et al 2025
JDW 684	pDest-I-Sce-R4-R3	attR4-R3 gate with SV40 polyA flanked by Tol2 inverted repeats	Multisite Cloning, I-Sce zebrafish transgenesis		Danio rerio	Wythe Lab / Gillespie et al 2025
JDW 1186	pAAV-Tet-Off-FLEX-WPRE-pA	AAV, Cre-dependent expression of insert, Tet-Off, with EFS driving destabilized tTA Destination vector	AAV, Tet-Off	Figure 7	viral	Wythe Lab / Gillespie et al 2025

Development • Supplementary information

Table S6. Novel expression vectors

Plasmid ID #	Name	Description	Uses	Figure	Origin Species	Species for Use	Lab Origin / Citation
JDW 1477	pDEST-EF1a-EGFP-mm-miR-500	EF1a driven expression of EGFP and murine miR-500	Constitutive expression of microRNA 500 in mammalian cells		Ef1a = homo sapien miR 500 = mus musculus	Mammalian cells	Wythe Lab / Gillespie et al 2025
JDW 1478	pDEST-EF1a-EGFP-miR-MCS	EF1a driven expression of EGFP with an empty intron	Constitutive expression of EGFP in mammalian cells		Ef1a = homo sapien	Mammalian cells	Wythe Lab / Gillespie et al 2025
JDW 1415	pDEST-EF1a-EGFP-hs-miR-126	EF1a driven expression of EGFP and human miR-126			Ef1a = homo sapien miR 126 = homo sapien	Mammalian cells	Wythe Lab / Gillespie et al 2025
JDW 410	pCAGEN-rtTA-IRES-nls-EGFP-WPRE-bGHpA	CAG driven rtTA (Tet-On) followed by IRES and GFP	Constitutive expression of Tet transactivator and GFP in mammalian cells	Figure 5	CAGGS = Synthetic; CMV (viral), b-actin (chicken), b-globin (rabbit) EGFP = Synthetic; Aequorea victoria	Mammalian cells	Wythe Lab / Gillespie et al 2025
JDW 1500	pCAGEN-nls-BFP-FLAG-WPRE-pA	CAG driven 3xNLS-mTagBFP-FLAG-bGH-pA	Constitutive expression of nls-BFP-FLAG in mammalian cells	Figure S3	CAGGS = Synthetic; CMV (viral), b-actin (chicken), b-globin (rabbit) BFP = Synthetic; Entacmaea quadricolor	Mammalian cells	Wythe Lab / Gillespie et al 2025
JDW 453	pCS-Phi31o	CMV driven Phi31 recombinase	Constitutive expression of Phi31 in mammalian cells or SP6 driven in vitro transcription of Phi31 mRNA		CMV - viral	In vitro transcription, mammalian cells	Wythe Lab / Gillespie et al 2025

Development • Supplementary information

JDW 721	pAAV-TRE-FLEX-Lifeact-mScarlet-HA-WPRE-EFS-d2tTA	AAV vector for Cre-dependent expression of insert, Tet-Off (destabilized).	Cre-dependent, Tet-Off expression of F-actin red fluorescent reporter in mammalian cells and mice		NA	Mammalian cells, mice	Wythe Lab / Gillespie et al 2025
JDW 1129	pCAGEN-Luciferase-P2A-H2A-mCherry	CAG driven luciferase and nuclear mCherry (From JDW 471)	Constitutive expression of luciferase and nuclear localized mCherry in mammalian cells	Figure 5	CAGGS = Synthetic; CMV (viral), b-actin (chicken), b-globin (rabbit) Luciferase = Photinus pyralis mCherry = Synthetic; Discosoma sp.	Mammalian cells	Wythe Lab / Gillespie et al 2025
JDW 945	pB-Tet-On 3.0:: Luc-P2A-H2A-mCherry	Tet-on, NEO selectable, expression of luciferase and nuclear mCherry (from JDW 931)	*piggyBac* ITR flanked Tet On vector driving luciferase and nuclear localized mCherry	Figure 7	Luciferase = Photinus pyralis mCherry = Synthetic; Discosoma sp.	Mammalian cells, mice	Wythe Lab / Gillespie et al 2025
JDW 1130	pB-Tet-On 2.0:: Luc-P2A-H2A-mCherry	Tet-on, NEO selectable, expression of luciferase and nuclear mCherry (from JDW 1086)	*piggyBac* ITR flanked Tet On vector driving luciferase and nuclear localized mCherry	Figure 7	Luciferase = Photinus pyralis mCherry = Synthetic; Discosoma sp.	Mammalian cells, mice	Wythe Lab / Gillespie et al 2025
JDW 1154	pB Tet-On::Luc-P2A-H2A-mCherry	Tet-on, NEO selectable, expression of luciferase and nuclear mCherry (from pB Tet-On, Addgene)	*piggyBac* ITR flanked Tet On vector driving luciferase and nuclear localized mCherry	Figure 7	Luciferase = Photinus pyralis mCherry = Synthetic; Discosoma sp.	Mammalian cells, mice	Wythe Lab / Gillespie et al 2025
JDW 970	pB-Tet-Off-FLEX-Luc-P2A-H2A-mCherry	Cre-dependent, Tet-Off Luciferase and mCherry (from JDW 936)	*piggyBac* ITR flanked, Cre-dependent, Tet-Off vector driving luciferase and nuclear localized mCherry	Figure 7	Luciferase = Photinus pyralis mCherry = Synthetic; Discosoma sp.	Mammalian cells, mice	Wythe Lab / Gillespie et al 2025

JDW 1404	pB-CAG-lsl-mScarlet-V5	Cre-dependent, CAG driven expression of mScarlet	*piggyBac* ITR flanked Cre recombinase switch RFP reporter for stable integration		CAGGS = Synthetic; CMV (viral), b-actin (chicken), b-globin (rabbit) mScarlet = Synthetic	Mammalian cells, mice	Wythe Lab / Gillespie et al 2025
JDW 1131	pCAGEN-Dre-nls	CAG driven Dre recombinase	Constitutive expression of Dre recombinase in mammalian cells	Figure 6	CAGGS = Synthetic; CMV (viral), b-actin (chicken), b-globin (rabbit)	Mammalian cells	Wythe Lab / Gillespie et al 2025
JDW 486	pCAGEN-DreERT2	CAG driven Dre recombinase fused to mutant ERT2	Constitutive expression of tamoxifen-inducible Dre recombinase in mammalian cells		CAGGS = Synthetic; CMV (viral), b-actin (chicken), b-globin (rabbit)	Mammalian cells	Wythe Lab / Gillespie et al 2025
JDW 35	pMEF2c-AHF-Dre-nls	Mef2c AHF/F6 enhancer driving expression of Dre recombinase	Anterior heart field (AHF)-specific expression of Dre recombinase in mice		mus musculus	Mice	Devine et al., 2014
JDW 196	pMEF2c-AHF-DreERT2	Mef2c AHF/F6 enhancer driving expression of Dre-ERT2 mutant fusion	Anterior heart field (AHF)-specific expression of tamoxifen-inducible Dre recombinase in mice		mus musculus	Mice	Devine et al., 2014
JDW 473	pCAGGS-Rox-nlsKate5V-stop-Rox-myr-Flag-BFP-NEO	CAGGS driven rox nuclear mKate2, myristoylated mTagBFP switch reporter for Dre recombinase	Dre recombinase-dependent fluorescent RFP to BFP switch reporter	Figure 6	CAGGS = Synthetic; CMV (viral), b-actin (chicken), b-globin (rabbit)	Mammalian cells, mice	Wythe Lab / Gillespie et al 2025
JDW 694	pCS2-Sun1-2xsfGFP-6xMYC-pA	CMV driving expression of the nuclear envelop protein Sun1 fused to two copies of sfGFP with 6 MYC tags.	External nuclear envelop EGFP and MYC affinity tag	Figure 1	CMV - viral	Mammalian cells	Wythe Lab / Gillespie et al 2025

Development • Supplementary information

JDW 1353	pCAGEN-Vhh-Lamin-mNeonGreen-myr-BFP	Lamin nanobody fused to mNeonGreen IRES myr-mTagBFP	Nanbody against a nuclear envelop protein, fused to green fluorescent reporter with a downstream IRES and cell membrane BFP reporter		CAGGS = Synthetic; CMV (viral), b-actin (chicken), b-globin (rabbit)	Mammalian cells	Wythe Lab / Gillespie et al 2025
JDW 1308	pCAGEN-Actin-Vhh-mNeonGreen-HA-pA	Lamin nanobody fused to mNeonGreen tagged with an HA	Nanbody against a nuclear envelop protein, fused to green fluorescent reporter	Figure S3			Wythe Lab / Gillespie et al 2025
JDW 1326	pCAGEN-nls-mCherry-IRES-myr-EGFP	NLS-mCherry followed by IRES and membrane EGFP	Constitutive expression of nuclear mCherry and cell membrane EGFP	Figure S3	CAGGS = Synthetic; CMV (viral), b-actin (chicken), b-globin (rabbit)	Mammalian cells	Wythe Lab / Gillespie et al 2025
JDW 1233	pTol2-Hsp70-lsl-H2B-mCerulean-mScarlet; fli1ep-zCreI-BFP	Tol2 based vector with endothelial-driven Cre	Heat shock inducible, nuclear localized mCerulean to RFP Cre/lox switch reporter with an endothelial promoter driving Cre and BFP in the opposite direction		Danio rerio	Zebrafish	Wythe Lab / Gillespie et al 2025
JDW 1234	pTol2-fli1-lsl-AmCyan-mScarlet; hsp70-zCreI-BFP	Expression of fli1 driven loxP flanked AmCyan followed by mScarlet-I with hsp70-TagBFP-zCre in opposite direction	Endothelial AmCyan to RFP Cre/lox switch reporter with heatshock promoter driving BFP tagged Cre in the backbone		Danio rerio	Zebrafish	Wythe Lab / Gillespie et al 2025
JDW 1235	pTol2-Ubi-lsl-EGFP-nls-mCherry; hsp70-zCreI-BFP	Expression of Ubi driven loxP flanked GFP followed by nls-mCherry reporter with hsp70-TagBFP-zCre in	Ubiquitous EGFP to nuclear mCherry Cre/lox switch reporter with heatshock promoter driving Cre-BFP fusion in	Figure 8	Danio rerio	Zebrafish	Wythe Lab / Gillespie et al 2025

Development • Supplementary information

		opposite direction	backbone				
JDW 1057	pTol2-Ubi-lsl-EGFP-nls-mCherry-Hsp70-zCreI-TagBFP2-d2-2xins-	Expression of Ubi driven loxP flanked GFP followed by nls-mCherry reporter with hsp70-driving destabilized TagBFP-zCre in opposite direction	Ubiquitous EGFP to nuclear mCherry Cre/lox switch reporter with insulators downstream of heatshock promoter driving Cre-BFP fusion in backbone	Figure 8	Danio rerio	Zebrafish	Wythe Lab / Gillespie et al 2025
JDW 1236	pTol2-Ubi-lsl-EGFP-nls-mCherry; fli1-zCreI-BFP	Expression of Ubi driven loxP flanked GFP followed by nls-mCherry reporter with fli1 driving TagBFP-zCre in opposite direction	Ubiquitous EGFP to nuclear mCherry Cre/lox switch reporter with endothelial-specific promoter driving Cre-BFP fusion in backbone		Danio rerio	Zebrafish	Wythe Lab / Gillespie et al 2025
JDW 1316	pTol2-Ubi-lexA-lexO-nls-GFP-pA	Tol2 based vector for RU486 inducible expression of GFP, 1st generation	Ubiquitous LexA transactivator and LexO driven nls-GFP reporter for RU5486 inducible expression	Figure 4	Synthetic	Zebrafish	Wythe Lab / Gillespie et al 2025
JDW 1381	pTol2-Ubi-dlexA-lexO-nls-GFP-pA -nls-GFP-pA	Tol2 based vector for RU486 inducible GFP expression with a destabilized lex transactivator	Ubiquitous destablized LexA transactivator and LexO driven nls-GFP reporter for RU5486 inducible expression	Figure 4	Synthetic	Zebrafish	Wythe Lab / Gillespie et al 2025
JDW 1382	pTol2-Ubi-dlexA-lexO-c-fos-nls-GFP-pA	Tol2 based vector for RU486 inducible GFP expression a destabilized lex transactivator and c-fos	Ubiquitous destablized LexA transactivator and LexO-c-fos minimal promoter driven nls-GFP reporter for RU5486 inducible	Figure 4	Synthetic	Zebrafish	Wythe Lab / Gillespie et al 2025

Development • Supplementary information

			expression				
JDW 1402	pCAGEN-FastFUCCI-P2A-H2A-tdiRFP	FastFUCCI reporter followed by H2A tdiRFP	For visualizing cell cycle in mammalian cells		CAGGS = Synthetic; CMV (viral), b-actin (chicken), b-globin (rabbit)	Mammalian cells	Wythe Lab / Gillespie et al 2025
JDW 1494	pCAGEN-FastFUCCI	CAG driven expression of monomeric Kusabira-Orange 2(mKO2) fused to human Cdt1(aa 30-120) followed by a T2A cleavage peptide and mAzamiGreen fused to human Geminin (aa 1-110)	For fluorescent monitoring of cell cycle state in mammalian cells	Figure 2, Figure S4	CAGGS = Synthetic; CMV (viral), b-actin (chicken), b-globin (rabbit)	Mammalian cells	Wythe Lab / Gillespie et al 2025
JDW 1489	pTol2-Ubi-zFucci-pA; exorh-GFP	Tol2 flanked ubiquitin promoter driven expression of mCerulean fused to zebrafish Geminin followed by a P2A cleavage peptide and mCherry fused to zebrafish Cdt1, with pineal gland driven GFP reporter	Ubiquitous cell cycle reporter (zFucci) for Cerulean/mCherry monitoring of cell cycle state	Figure 2	Danio rerio	Zebrafish	Wythe Lab / Gillespie et al 2025
JDW 1495	pLenti-BLAST-CMV-LiCre-WPRE	CMV driven expression of LiCre along with SV40 driven expression of Blasticidin	For expression of bluelight inducible Cre recombinase	Figure 3	Synthetic	Mammalian cells, mice	Wythe Lab / Gillespie et al 2025

Development • Supplementary information

JDW 1369	pCAGEN-hyPBase-WPRE	CAGGS promtoer driven hyperactive PBase	For *piggyBac* transposon-based integration of cargo plasmids		CAGGS = Synthetic; CMV (viral), b-actin (chicken), b-globin (rabbit)	Mammalian, mouse	Wythe Lab / Gillespie et al 2025
JDW 1166	pGLAST-hyPBase-WPRE	GLAST/EEAT1 promoter (radial glia) driving hyperactive PBase	For *piggyBac* transposon-based integration of cargo plasmids	Figure 8	Glast = homo sapien	Mammalian, mouse	Wythe Lab / Gillespie et al 2025
JDW 1502	pCAGEN-FastFUCCI-IRES-myr-mTagBFP	CAG driven expression of monomeric Kusabira-Orange 2(mKO2) fused to human Cdt1(aa 30-120) followed by a T2A cleavage peptide and mAzamiGreen fused to human Geminin (aa 1-110) followed by an IRES myr-mTagBFP	For fluorescent monitoring of cell cycle state in mammalian cells.	Figure 2, Figure S4	CAGGS = Synthetic; CMV (viral), b-actin (chicken), b-globin (rabbit)	Mammalian, mouse	Wythe Lab / Gillespie et al 2025
JDW 1503	pCAGEN-FastFUCCI-IRES-myr-mKate2	CAG driven expression of monomeric Kusabira-Orange 2(mKO2) fused to human Cdt1(aa 30-120) followed by a T2A cleavage peptide and mAzamiGreen fused to human Geminin (aa 1-110) followed by an IRES myr-mKate2	For fluorescent monitoring of cell cycle state in mammalian cells.		CAGGS = Synthetic; CMV (viral), b-actin (chicken), b-globin (rabbit)	Mammalian, mouse	Wythe Lab / Gillespie et al 2025

Development • Supplementary information

JDW 1511	pB-EF1a-FastFUCCI-IRES-3xnls-mTagBFP2	EF1a driven expression of monomeric Kusabira-Orange 2(mKO2) fused to human Cdt1(aa 30-120) followed by a T2A cleavage peptide and mAzamiGreen fused to human Geminin (aa 1-110) followed by an IRES myr-mTagBFP	A *piggyBac* ITR flanked reporter for monitoring cell cycle states in mammalian cells. For stable integration	Figure 2, Figure S4	Ef1a = homo sapien	Mammalian, mouse	Wythe Lab / Gillespie et al 2025
JDW 1515	pB-EFS-FastFUCCI-IRES-3xnls-mTagBFP2	EFS driven expression of monomeric Kusabira-Orange 2(mKO2) fused to human Cdt1(aa 30-120) followed by a T2A cleavage peptide and mAzamiGreen fused to human Geminin (aa 1-110) followed by an IRES myr-mTagBFP	A *piggyBac* ITR flanked reporter for monitoring cell cycle states in mammalian cells. For stable integration	Figure 2, Figure S4	EFS = homo sapien	Mammalian, mouse	Wythe Lab / Gillespie et al 2025
JDW 1516	pTol2-Crestin-zFUCCI-IRES-EGFP-caax	Tol2 flanked crestin promoter driven expression of mCerulean fused to zebrafish Geminin followed by a P2A cleavage peptide and mCherry fused to zebrafish Cdt1, followed by an IRES	Cell cycle reporter (zFucci) for Cerulean/mCherry monitoring of cell cycle state in neural crest cells		Crestin = Danio rerio	Zebrafish	Wythe Lab / Gillespie et al 2025

Development • Supplementary information

		EGFP-caax					
JDW 1517	pTol2-Fli1-zFUCCI-IRES-mTagBFP2	Tol2 flanked fli1 promoter driven expression of mCerulean fused to zebrafish Geminin followed by a P2A cleavage peptide and mCherry fused to zebrafish Cdt1, followed by an IRES mTagBFP2	Cell cycle reporter (zFucci) for Cerulean/mCherry monitoring of cell cycle state in endothelial cells		fli1 = Danio rerio	Zebrafish	Wythe Lab / Gillespie et al 2025
JDW 1528	pTol2-Unc-Actin-mCherry-IRES-StayGold-pA	Tol2 flanked Unc promoter driving expression of mCherry fused to actin followed by an IRES-StayGold reporter.	Labels muscular actin in zebrafish		Unc = danio rerio	zebrafish	Wythe Lab / Gillespie et al 2025
JDW 1529	pCAGEN-H2B-V5-mScarlet3-S2	CAGGS driven mScarlet3-S2 to label nuclei	labels nuclei		CAGGS = Synthetic; CMV (viral), b-actin (chicken), b-globin (rabbit)	Mammalian, mouse	Wythe Lab / Gillespie et al 2025
JDW 1530	pCAGEN-H2B-V5-mScarlet3-H	CAGGS driven mScarlet3-H to label nuclei	labels nuclei	Supplemental Fig. 3	CAGGS = Synthetic; CMV (viral), b-actin (chicken), b-globin (rabbit)	Mammalian, mouse	Wythe Lab / Gillespie et al 2025
JDW 1532	pCAGEN-ActinVhh-sfGFP-P2A-HA-tdiRFP-caax	CAGGS driven sfGFP to label actin followed by a cleavage peptide and a tdiRFP that labels the cell membrane.	Labels actin and cell membrane		CAGGS = Synthetic; CMV (viral), b-actin (chicken), b-globin (rabbit)	Mammalian, mouse	Wythe Lab / Gillespie et al 2025

Development • Supplementary information

Dataset 1. Empty plasmid database for free use

Available for download at
https://journals.biologists.com/dev/article-lookup/doi/10.1242/dev.204308#supplementary-data

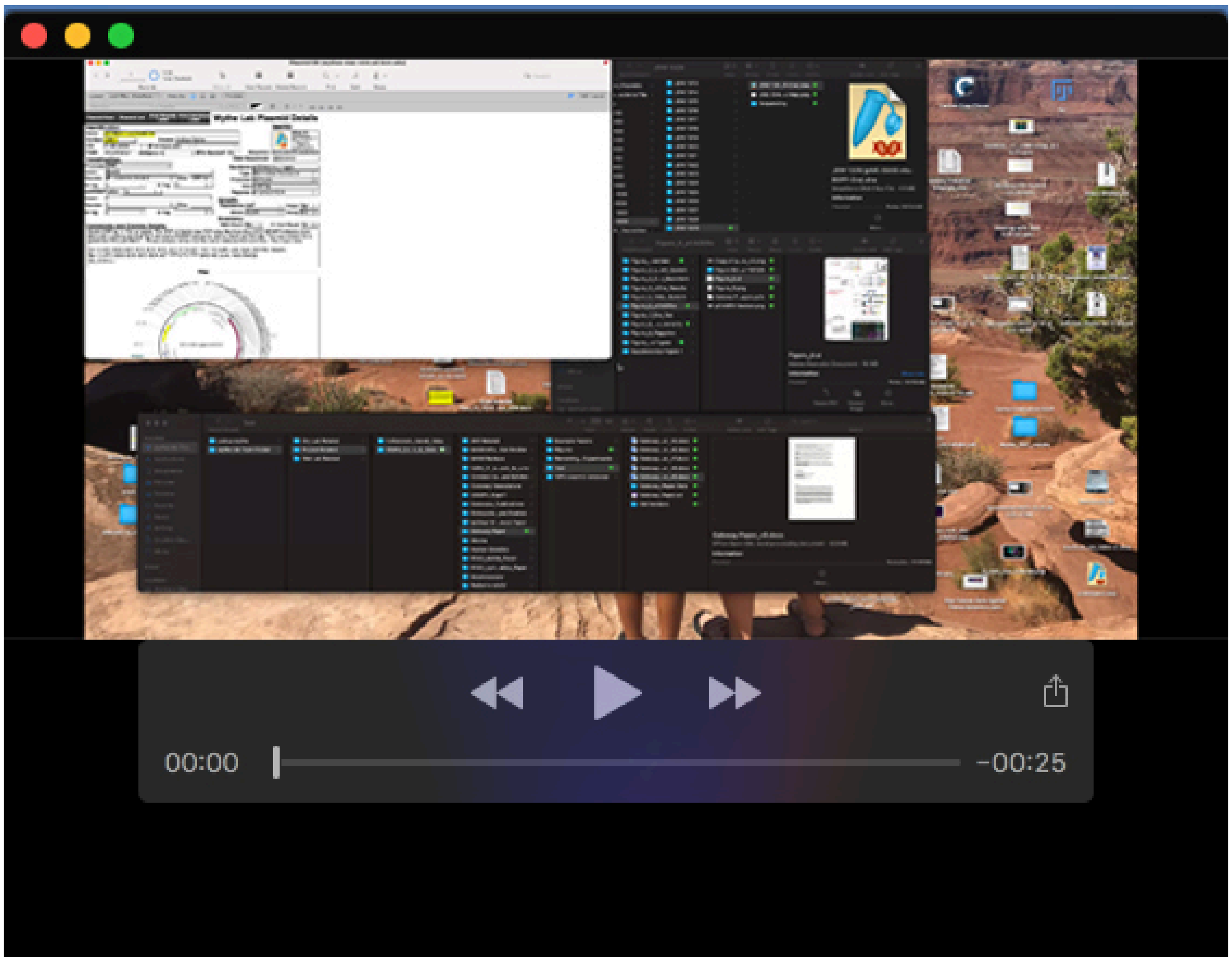

Movie 1. A Video Tutorial for Entering a Plasmid. From the “applications” dropdown list or home menu, selecting the plasmid database will open the default record view. Expand the window and zoom in. Go to “Create New Record” and then begin entering your record’s info, starting with the numbering system your lab will employ. Select the typical “use” categories, then the backbone and type of plasmid, and enter the growth conditions and other information, such as the construction, inserts, tags, and other relevant information. Then plasmid maps and electronic files can be added to the record directly by dragging and dropping these files into the database. Finally, add any relevant cloning for further information on the design and construction of this plasmid.

Development • Supplementary information

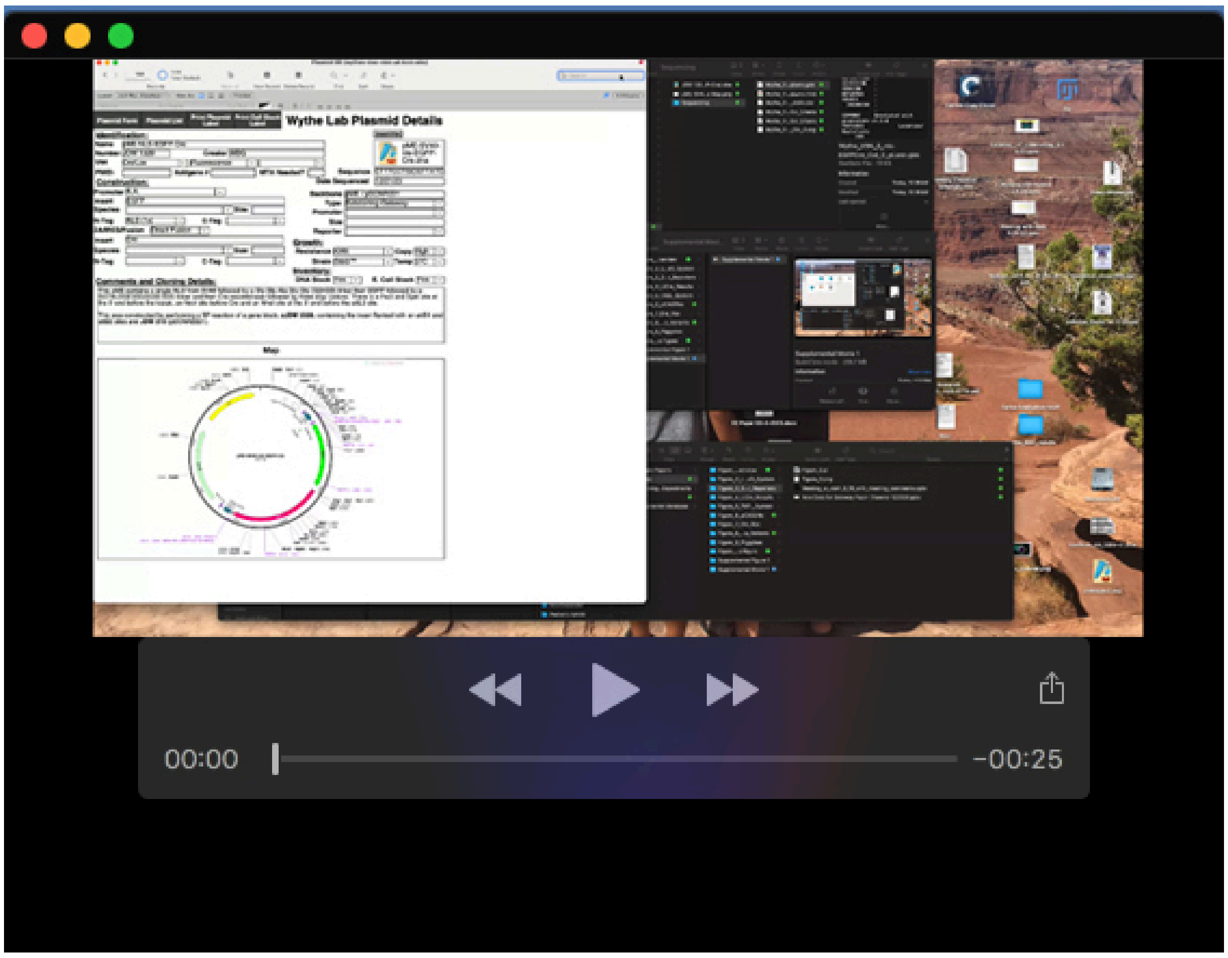

Movie 2. Navigating the Plasmid Database. To find any plasmids that meat specific criteria, one can use the upper right hand search window and entering your query term (in this case, "Cre"). We suggest then going to the Layout menu and selecting "List View" as this is more manageable and all records that contain this term can be easily scrolled through and visualized simultaneously, rather than in the individual record view. In our database, we highlight plasmids that we cannot find glycerol stocks or DNA for, as these need to be found or replaced. When you find a plasmid that may be of interest, select it, then tap the "plasmid form" tab at the top, and it will bring up the record view. You can then enter another search term, such as "pME" to view every pME (middle entry) plasmid and return to list view. We would also note that using a Dymo label printer, we use the database to print plasmid labels for the side and tops of Eppendorf tubes (1.7 mL) to eliminate poor labelling and misidentification of plasmids and e. coli stocks. When you chose this option, ensure you select only the record being viewed (rather than the entire database).

Supplementary Materials and Methods

p5E Construct Generation:

p5E-human EF1α (JDW 1164) was generated by digesting p5E-MCS (Chien Lab plasmid #228) (a kind gift of the late Dr. Chi-Bin Chien, University of Utah) with HindIII and BamHI. The human EF1α promoter was subcloned using the same sites from pAAV-oChIEF-P2A-TdTomato-WPRE-bGH-A (Addgene # 51094).

p5E-EFS (JDW 1319) contains the core promoter of human EF1α (*Elongation Factor-1α*) (also known as EF-1α short, or EFS) lacking the downstream intron for lower transcriptional activity based on Addgene #60224 (Platt et al., 2014). A gene block with a pause site from human *alpha 2 globin* to prevent transcriptional read through upstream of EFS, with flanking 5' attB4 and 3' attB1 sites was synthesized and then BP recombined into pDONR P4-P1r.

p5E CAG (JDW 912) was created by inserting the synthetic CAGGS promoter into p5E-MCS (Chien Lab plasmid #228) via KpnI to BamHI.

A gene block containing ~1.2 kb spanning the promoter (-1225 up to -6) of the human *UBIQUITIN* locus was synthesized with flanking 5' attB4 and 3' attB1 sites and cloned via BP reaction into pDONR P4-P1r to generate p5E-hs_UbiC-pro (JDW 1461).

p5E-ICAM2 full length (JDW 1047) contains the 397-bp human *ICAM2* promoter amplified by PCR using a geneblock template designed based on Addgene plasmid #99736 (Wang et al., 2016). XhoI and SacII sites, as well as attB4 and attB1 sequences, were added by the following primers: oJDW 1776 5'-(XhoI) and oJDW 1777 5'-(SacII). The purified PCR product was digested and inserted into p5E-MCS (Chien lab plasmid #228)(Kwan et al., 2007) using the same restriction sites.

p5E-ICAM2 (JDW 1046) contains only the minimal 140-bp promoter, which was amplified by PCR using JDW 1047 as a template and the following oligos: oJDW 1774 (FWD XhoI): 5'-aaaaCTCGAGGTAGAACGAGCTGGTGCACGTGGC, oJDW 1775 (REV SacII): 5'-aaaaCCGCGGCCAAGGGCTGCCTGGAGGGAG. The purified PCR product was digested and inserted into p5E-MCS (Chien lab plasmid #228)(Kwan et al., 2007) using the same restriction sites.

p5E-*ICAM2-c-Fos* (JDW 1117) was generated by performing a BP reaction between a gene block (Twist Bioscience) containing the 140-bp minimal human *ICAM2* promoter upstream of the 93-bp murine *c-Fos* minimal basal promoter (5'-CCAGTGACGTAGGAAGTCCATCCATTCACAGCGCTTCTATAAAGGCGC CAGCTGAGGCGCCTACTACTCCAACCGCGACTGCAGCGAGCAACT-3'), flanked by *attB4* and *attB1* sites, which was BP recombined into pDONR P4-P1r.

The human *CLDN5* promoter and chimeric intron was generated by PCR using Addgene plasmid #29299 (pEMS1503) (de Leeuw et al., 2014) as a template and the following oligos: oJDW 1948 (CLDN5 FWD + Sal I) 5'-aaaaGTCGACAACCCTTAATGAATTCGAGCTCCTAGGC and oJDW (1949 CLDn5 REV + HindIII) 5'-aaaaAAGCTTTTTGAAGAATAGGAACTTCGGAATAGGAA CTTCC. The resulting amplicon was digested with SalI and HindIII, then cloned into p5E-MCS (Chien Lab plasmid #228) using the same sites to generate p5E *CLDN5* (JDW 1120).

p5E *CDH5* (JDW 1239) was generated by PCR amplification of a 2,486 bp fragment and 15 bp of an exon from murine genomic DNA using the following primers containing a 5' attB4 and 3' attB1 site: FWD, 5'-ggggACAACTTTGT ATAGAAAAGTTGCTCGAGGTCGACt CTAGTAGCAGAAACAAGG, REV, 5'-ccggAGCCTGCTT TTTTGTACAAACTTGAGAA TTCAGGGCCGAGCTTTGTGGAGAGCAC. The resulting amplicon was BP recombined into pDONR P4-P1r.

The 544 bp human *Troponin T* (*TNNT2*) promoter was amplified from an existing template using primers containing a 5' flanking attB4 and 3' attB1 site: FWD 5'-GGGGACAACTTTGTATAGAAAAGTTGaaagcttCTCAGTCCATTAGGAGC and REV 5'-ccggAGCCTGCTTTTTTGTACAAACTTGAaccggtGAATTCCTGCCGACAGATCCTGG and then recombined with pDONR P4-P1r (p5E Donor) to make a mammalian pan-cardiomyocyte promoter clone p5E-*TNNT2* (JDW 1208).

The human *GLAST* promoter was cloned by PCR using primers that contained a 5' attB4 and a 3' attB1 site: FWD, 5'- ggccCAACTTTGTATAGAAAAGTTGATCGAT AGGTACCATGTCTACACAAACtg, REV, 5'-AGCCTGCTTTTTTGTACAAACTTGACTAGTccggt GGATCTCGAGCCCatcaagc. The resulting amplicon was purified by PCR Cleanup (Qiagen) then recombined with pDONR P4-P1r in a BP reaction to generate p5E Glast (JDW 1182).

p5E-TetO$_{(8x)}$-CMV$_{min}$ (JDW 1087) contains a 2nd generation Tet response element (TRE) composed of eight 19-bp *tet* operator sequences located upstream of a 104-bp minimal CMV promoter (Agha-Mohammadi et al., 2004, Kim et al., 2016b). This was created by subcloning a Tet-Response-Element containing 8 Tet Operons (Tet Response Element or TREs) upstream of a minimal CMV promoter from JDW 931 (pB_Tet-On_DEST_EFS_mODC_rtTA_IRES_NEO) into p5E MCS (Chien Lab plasmid #228) via digestion with PspXI and SacII followed by a T4 DNA Ligase reaction and transformation in Stbl3 bacteria.

p5E-TRE3GV (JDW 1163) contains a 3rd generation TRE promoter composed of seven 19-bp *tet* operator sites upstream of a 12-bp pTight hybrid sequence(Clontech, 2003) followed by a modified 68-bp minimal CMV promoter that contains a synthetic TFIIB binding site and TATA box(Lagrange et al., 1998), which together ensure low basal activity and

Development • Supplementary information

maximal responsiveness to doxycycline and high performance with lentivirus and retrovirus (Loew et al., 2010). This was created by digesting JDW 447 (pBT346.6-TRE3G-FRT) with XhoI and HindIII and the resulting fragment was inserted via the same restriction sites into p5E-MCS (Chien Lab plasmid #228).

For p5E-LexAOp-c-fos (JDW 1254), we exchanged several components to optimize the original design(Emelyanov and Parinov, 2008). The original construct had an SV40 polyA followed by a terminator sequence from nopaline synthase (NOS) in *Agrobacterium tumefaciens* and then 4 colE1 sites (a lexA operator) and a minimal 35S promoter from cauliflower mosaic virus (CaMV 35S promoter). In our newer iteration the SV40 polyA signal is followed by an RNA Polymerase II transcriptional pause site from human *alpha-2 globin* to prevent transcriptional read through from an upstream promoter, followed by the synthetic lexA operator consisting of six ColE1 operator sites (rather than four) and the mouse *c-Fos* minimal promoter to prevent basal transcriptional activity (Dorsky et al., 2002, Scott and Baier, 2009).

For a zebrafish, pan-cardiomyocyte promoter, a ~900 bp promoter (including exon 1) of zebrafish *myl7* (also known as cardiac myosin light chain 2, or *cmlc2*) was designed based on the sequence from pTol2DestCG2 (Kwan et al., 2007). Flanking flanking attB4 and attB1 sites were added to this element and the resulting geneblock was BP recombined with pDONR P4-P1r to generate p5E-*myl7-pro* (JDW 1148).

The 503 bp, muscle specific *unc503* promoter spanning -600 to -98 of the zebrafish *unc-45b* locus (Berger and Currie, 2013), was synthesized as part of a gene block (Twist Biosciences), followed by a murine *c-Fos* minimal promoter and a rabbit *beta-globin* intron to facilitate robust muscle expression, flanked by *attB4* and *attB1* sites at the 5' and 3' end, respectively, was synthesized as a geneblock and then recombined into pDONR P4-P1r by a BP Clonase reaction to generate p5E-*Unc-cFos-B-Globin* (JDW 1365).

p5E-Crestin (JDW 1320) contains an 844 bp element from a previously described ~1 kb neural crest-specific enhancer from zebrafish *crestin*(Kaufman et al., 2016) followed by a minimal murine *c-Fos* promoter, then a 652 bp rabbit *beta globin* intron. This element was synthesized as a geneblock with flanking *attB4* and *attB1* sites and then inserted into pDONR P4-P1r by BP recombination.

p5E-*Dll4-F2-E1b-b-globin* (JDW 1366) contains the murine *Dll4* F2 arterial specific enhancer upstream of a minimal E1b promoter and intron for arterial and endocardial expression (Wythe et al., 2013). This construct was generated by BP recombining a gene block containing the murine Dll4 F2 enhancer followed by a minimal E1b promotor and intron all flanked by attB4 and attB1 sites with pDONR P4-P1r.

To construct p5E-*5xC120-c-Fos* (JDW 1241), a cassette containing a SV40 polyA followed by a transcriptional pause site, a NOS terminator, then 5x C120 sites upstream of

Development • Supplementary information

the murine *c-Fos* minimal basal promoter was amplified from JDW 1109 (pME-TAEL-c-Fos) using the following primers: oJDW 2185 (p5E FWD) (66-mer):5'-ggggACAACTTTGTATAGAAAAGTTGGAATTCTAGCAGACATGATAAGATACATTGATGAGTTTGG and oJDW 2186 (p5E REV) (58-mer): 5'-ccggAGCCTGCTTTTTTGTACAAACTTGATCTAGAACTAGTGCGGCCGCGTTAGTTGC, and the resulting 5' attB4 and 3' attB1 flanked PCR product was BP recombined into pDONR P4-P1.

To create p5E-*MCS-c-Fos-beta-globin* (JDW 1237), a Geneblock fragment was ordered with attB4 and attB1 flanking sites with a multiple cloning site (5'-KpnI, XhoI, HindIII, SpeI, BglII, PacI, SalI, BamHI, PmeI) upstream of the murine c-Fos minimal promoter followed by a rabbit beta-globin intron, followed by AgeI, ClaI and SnaBI sites.

All p5E clones were confirmed by Sanger sequencing.

pME Construct Generation:

Unless stated otherwise, all middle Entry (pME) vectors were produced by PCR amplification of the desired template using attB1/B2-flanked primers, or direct synthesis of the fragment with flanking attB1/B2 sites, followed by a BP reaction with pDONR221 (Invitrogen). In some cases, a modified pDONR221 that we created which contains a novel multiple cloning site (EcoRI-SalI-BamHI-KpnI-SmaI-NotI-XhoI-EcoRI), known as pME-MCS (JDW 455), was used for restriction enzyme-based cloning. The inserts within these pME vectors usually contain a consensus mammalian Kozak sequence (Kozak, 1987) (5'-GCCACC-3') for optimal ribosome binding and initiation of translation upstream of the first start codon (ATG). Unless otherwise noted, all middle Entry clones contain a stop codon at the 3' end and thus cannot be used for N-terminal fusions to a protein of interest by placing them upstream of a p3E clone. In cases where middle Entry clones contain multiple inserts, the coding regions of unique cDNAs are separated by a viral 2A peptide (2A). The use of a 2A-generated ribosomal skip event should ensure more equivalent protein production of independent polypeptide inserts, unlike the use of an internal ribosomal entry site (IRES), which is less likely to generate equal expression levels of the downstream insert relative to the upstream cassette(Donnelly et al., 2001, Kwan et al., 2007, Mizuguchi et al., 2000, Yu et al., 2003).

pME-MCS-WPD (JDW 455) was construct by annealing two oligos into a double stranded duplex then recombining the dsDNA into pDONR221 via BP reaction to generate a novel pME-MCS vector with a multiple cloning site consisting of EcoRI-SalI-BamHI-KpnI-SmaI-NotI-XhoI-EcoRI flanked by *attL1* and *attL2* sites.

pME-V5-mTagBFP2 (JDW 830) contains an attL1/L2 flanked insert with BamHI, SpeI, and NcoI upstream of a Kozak, followed by a V5 epitope tag then a Gly-Gly-Ala-Gly-

Development • Supplementary information

Gly (G_2AG_2) flexible linker, where the a Met-Val-Ser-Lys-Gly-Glu insertion replaces the first Met-Ser-Glu residues of mTagBFP2 (p.I174A) (Subach et al., 2011), followed by HindIII, PacI, a stop codon, AscI, XhoI, and BglII and was shuttled from an AAV2 CAG-V5-mTagBFP2-WPRE-SV40-pA (to be described elsewhere) into an existing pME clone via SpeI to AscI.

pME-AmCyan1 (JDW 1151) was created by amplifying AmCyan from the vector pME lox-AmCyan-stop-lox (Zhou et al., 2011)(a kind gift of Drs. Caroline and Geoff Burns at Boston Children's Hospital). The insert is flanked 5' by AgeI before the Kozak sequence and a XhoI site after the stop codon at the 3' end. The AmCyan insert with flanking *attB1/B2* sites was amplified by PCR using the following oligos: oJDW 2012 (pME AmCyan FWD) 5'-gggg*ACAAGTTTGTACAAAAAAGCAGGCa*ccggtCGCCA CCATGGCCCTG TCC, oJDW 2013 (pME AmCyan REV) 5'-cccc*ACCACTTTGTACAAGAAAG CTGGGc*tcgagCTTCAGAAGGGCACC. The resultant PCR product was purified (PCR Cleanup Kit, Qiagen) and cloned into pDONR21 by a BP reaction.

pME-mRuby2-3xMYC-stop (JDW 669) was created by amplifying the open reading frame, including a 5' Kozak, of mRuby2 followed by a 3x MYC tag and stop codon using an existing clone as a template (JDW 498, pUC57-KAN-FLEX-rtTA TetOn-deGradFP P2A mRuby2-3xMYC, to be described elsewhere) by PCR using the following primers: FWD-GGGGACAAGTTTGTACAAAAAAGCAGGCTGCCACCATGGTGTCTAaGGGCGAAGAGc and REV-GGG GAC CAC TTT GTA CAA GAA AGC TGG GTCTCAtcaggatctcaggtcctcc. The resulting PCR product was recombined with pDONR221 by BP reaction.

To create pME-V5-mScarlet-I (JDW 1323), a consensus Kozak sequence followed by a V5 epitope tag, then a Gly-Gly-Ala-Gly-Gly flexible linker, then mScarlet-I and an MCS followed by two stop codons, flanked by a 5' attB1 and 3' attB2 site, was synthesized (Twist Biosciences) and BP recombined into pDONR221.

pME-3xNLS-mTagBFP-WPRE-bGH-pA (JDW 484) was constructed by PCR sewing. Briefly, mammalian codon optimized 3xNLS-mTagBFP-FLAG, and a WPRE-bGH-polyA from Ai9 (Addgene #22799)(Madisen et al., 2010), were separately amplified by PCR using primers that contained a homologous, overlapping region in the 3' oligo of mTagBFP and the 5' oligo of the WPRE insert, respectively, as well as flanking sites. The two PCR products were then combined and amplified together using oligos at the 5' and 3' that contained attB1/B2 sites and the resulting amplicon was cloned into pDONR221 via a BP reaction. A similar strategy was used to generate pME-3xNLS-mKate-V5-WPRE-bGH-pA (JDW 485) and pME-3xNLS-EGFP-WPRE-bGH-pA (JDW 488).

To generate pME-nls-EGFP-WPRE-bGH-pA (JDW 488), an NLS-eGFP fragment and a WPRE-bgh-pA fragment from Ai9 (Addgene #22799, a kind gift of Dr. Hongkui Zeng), which were then stitched together and flanking attB1/attB2 sites were added using PCR

Development • Supplementary information

sewing. The entire cassette was then recombined into pDONR221 using a BP gateway reaction. The entire cassette can be excised by digestion with SwaI.

pME-nls-mKate2-V5-WPRE-bGH-pA (JDW 485) was created by amplifying an NLS-mKate2-V5 fragement and a WPRE-bgh-pA fragment from Ai9(Addgene #22799, a kind gift of Dr. Hongkui Zeng), which were then stitched together and flanking attB1/attB2 sites were added using PCR sewing. was then recombined into pDONR221 using a BP gateway reaction. The entire cassette can be excised by digestion with FseI.

pME-H2B-mCerulean (JDW 1150), was created by amplifying H2B-mCerulean from pME-*loxP*-H2B-mCerulean-stop-*loxP* (Harrison et al., 2015) (a kind gift of Drs. Michael Harrison and Ching-Ling Lien at Children's Hospital Los Angeles) using the following primers: oJDW 2010 (FWD) 5'-ggggga*CAAGTTTGTACAAAAAAGCAGGCT*CTAGAgaattcggtaccGCCACCATG CCAGAGCC and oJDW 2011 (Riedl et al.): 5'-ccccacc*ACTTTGTACAAGAAAGCTGGG*ctcgaggcggcc gcttaTTACTTGTACAGCTCGTCCATGCC. The purified PCR product was cloned into pDONR221 by BP reaction to generate pME-H2B-mCerulean. The attL1/L2 flanked insert contains 5' XbaI and KpnI sites upstream of a Kozak sequence followed by human Histone 1 H2Bj, then BamHI and AgeI restriction sites preceding mammalian codon optimized mCerulean(Rizzo et al., 2004), followed by two stop codons, then NotI and XhoI at the 3' end.

pME-H2B-V5-(n2)oxStayGold(c4) (JDW 1354) was generated by direct synthesis (Twist Biosciences) of a gene block composed of an attB1 site, SpeI, a consensus Kozak, human histone H2B.1, followed by a Gly-Gly-Ala-Gly-Gly linker, a V5 tag, an MCS with BamHI-AgeI-EcoRI sites, a Gly-Gly-Gly-Gly-Ser(x3) linker, KpnI, then (n2)oxStayGold(c4)v2.0, a stop codon, AscI, SalI, AccI, SnaBI, XbaI, attB2. This version of StayGold, engineered by Hirano and colleagues (Hirano et al., 2022), contains Cys174 and Cys208 mutated to Ile to render it usable in an oxidative environment. They also found the addition of nine amino acids from the N-terminal region of EGFP (n2) and ten amino acids at the C-terminus of dfGFP (c4) improved the targeting of StayGold to some subcellular components, hence the naming convention of (n2)oxStayGold(c4). Finally, version 2.0 is mammalian codon optimized for brighter, more stable expression(Hirano et al., 2022).

As StayGold is an obligate dimer, which may impact fusion protein function in vitro and in vivo, we also provide a monomeric variant engineered by Miyawaki and colleagues, mStayGold (also referred to as mStayGold(J) and StayGold QC2-6 FIQ) (Ando et al., 2024). To generate pME-H2B-mStayGold-WPRE (JDW 1384), an attB1/B2 flanked gene block containing BamHI, SpeI, human H2B, a BglII site, then then monomeric StayGold

Development • Supplementary information

(mStayGold), HindIII, WPRE was synthesized (Twist Biosciences) and recombined in pDONR221.

We provide another monomeric StayGold variant, mBaoJin, evolved by Subach and colleagues (S55T, H77R, E80G, Q140P, H141Q, C165Y, N171Y, T201A) that also contains the amino and carboxy terminus of mNeonGreen for added stability (Zhang et al., 2024). For comparison, an amino acid alignment of these variants is shown in **Fig. S11**. We fused the ORF of mBaoJin to H2B in a middle entry clone to create a nuclear localized, stable reporter (pME-H2B-mBaoJin-WPRE (JDW 1383)).

To generate pME-H2B-V5-mScarlet-3H (JDW 1513) a gene block flanked by attB1 and attB2 sites was designed containing human histone H2B followed by a V5 tagged mScarlet-3 variant, mYongHong (Chu et al., 2014). The insert was BP recombined with pDONR221. For comparison, an amino acid alignment of the mScarlet variants is shown in **Fig. S12**.

To generate pME-H2B-V5-mScarlet3-S2 (JDW 1514) a gene block flanked by attB1 and attB2 sites was designed containing human histone H2B followed by a V5 tagged mScarlet-3 variant, mScarlet3-S2 (Chu et al., 2014). The insert was BP recombined with pDONR221.

To generate pME-H2B-mCardinal (JDW 1418), a gene block containing a 5' attB1 site, and AgeI and BamHI flanking human histone H2B, followed by mCardinal (Chu et al., 2014), two stop codons, SpeI, and a 3' attB2 site was synthesized (Twist Biosciences) and recombined into pDONR221 by using BP clonase II.

To generate pME-Luc-P2A-H2A-mCherry (JDW 926), luciferase was amplified from JDW 842 (pB-CAG-EGFP-T2A-Luciferase) (a kind gift of Dr. Benjamin Deneen at Baylor College of Medicine) along with a BamHI and SpeI site and upstream Kozak consensus protein translation initiation sequence (GCC ACC ATG G) with primers oJDW 1368 (Luc FWD) 5'-GGGGggatccactagtgccaccATGGaagacgccaaaaacataaagaaagg and oJDW 1369 (Luc REV) 5'-CCCCCGGTACCcacggcgatctttccgcccttcttgG with a 3' in frame KpnI site (Gly-Thr-Gly). The resultant PCR amplicon and JDW 714 (pME-V5-KRAS-G12V-P2A-H2AZ/F-mCherry) were then digested with BamHI and KpnI, followed by a standard T4 DNA Ligation.

pME-Sun1-2xsfGFP-6xMyc-pA (JDW 681) was created by digesting pcDNA3.1_Sun1_2xsfGFP_6xMyc (a kind gift of Dr. Jeremy Nathans, Johns Hopkins University / HHMI)(Mo et al., 2015) with ClaI and PacI and inserting the resulting fragment into pDONR221.

pME LaminB-Vhh mNeonGreen-HA-pA (JDW 1312) encodes a camel nanobody (Vhh) targeting Lamin B (Rothbauer et al., 2006) fused to a fluorescent reporter. A gene block flanked by attB1/attB2 sites and containing a codon optimized Vhh anti lamin B (generated using VectorBuilder, https://en.vectorbuilder.com/) followed by a flexible linker (SLGGGGSGGGGSGGGGSGGGGSGT) and then mNeonGreen followed by a short flexible

Development • Supplementary information

linker (ASGSGGSG) and a single HA tag, then an SV40 polyA signal, was recombined into pDONR221 by performing a BP reaction.

pME-Golgi-mTagBFP2-HA-P2A-H2A-iRFP (JDW 1007) was synthesized by Gene Universal. JDW 1007 was constructed with an c-terminal linker containing NotI and XhoI restriction enzyme sites for the subsequent digestion and insertion of a second copy of iRFP to generate pME-Golgi-mTagBFP2-P2A-H2A-tdRFP (JDW 1079).

pME-myr-mTagBFP (JDW 1183) was generated by PCR addition of a 5' attB1 site upstream of a Kozak sequence and the eight amino acid myristoylation sequence from MARCKS (MGCCFSKT), which is sufficient to target proteins to the plasma membrane (Devine et al., 2014, Muzumdar et al., 2007) using the following primers: oJDW 2068 (attB1 FWD) 5'-TATCACAAGTTTGTACAAAAAAGCAGGCT and oJDW 2069 (attB2 REV) 5'-ATATCACCACTTTGTACAAGAAAGCTGGGT.

A middle entry clone for expressing an amino terminal construct at stoichiometric levels along with c-terminal F-actin mCherry reporter, pME P2A Lifeact mRuby2-3xMyc (JDW 678), was constructed by PCR using JDW 631 (pCRII-TOPO-MCS-P2A-mRuby2-3xMYC) as a template, with the following oligos: oJDW 838 5'-GGGGCAACTTTGTACAAAAAAGTTGCCGGAATTCGCCCTTGG and oJDW 839 5'-GGGGCAACTTTGTACAAAAAAGTTGCCGGAATTCGCCCTTGG. The resultant PCR product was BP recombined into pDONR221. This insert contains an attL1site, EcoRI 5' to the P2A, then a XmaI and SmaI site in the P2A, and an AvrII site for in frame cloning of new cDNAs, followed by the Lifeact peptide from yeast(Lam et al., 2012), a Gly-Asp-pro-Pro-Val-Ala linker with a BamHI-AgeI-NcoI then mRuby2 followed by 3x MYC tags, and a stop codon 5' to the attL2 site.

pME Lifeact mRuby2-3xMyc (JDW 1246) is a middle entry (pME) plasmid containing an amino terminal lifeact tag to bind to F-actin followed by mRuby2 and a c-terminal 3x MYC tag. The insert was amplified from JDW 631 (TOPO blunt Lifeact mRuby2 3xMyc) using oJDW 2147 5'-GGGGACAAGTTTGTACAAAAAAGCAGGCaaccggtggatccAGCCACCATGGGCGTGGCCGACTTGATCAAGAAGTTCG and oJDW 2148 5'- CCCCACCACTTTGTACAAGAAAGCTGGGcACTAGTACGCGTttactaggatctcaggtcctcctcgctgatcagc. Then resulting PCR product was then recombined into pDONR 221 (JDW 215) by a BP reaction.

To generate pME Lifeact mScarlet-I_3xHA (JDW 1247), Lifeact mScarlet-I 3xHA was amplified by PCR using JDW 713 (pME-V5-KRAS(G12V)-P2A-Lifeact-mScarlet-HA) as a template and oJDW 21955'-GGGGACAAGTTTGTACAAAAAAGCAGGCaaccggtggatccAGCCACCatgggcgtggccgacctgatcaagaagttcgagagcatcag, and oJDW 2196 5'-CCCCACCACTTTGTACAAGAAAGCTGGGcACTAGTACGCGTttaTTAAGCGTAATCTGGA

Development • Supplementary information

ACGTCATATGGATA. Then the insert was recombined into pDONR 221 (JDW 215) by a BP reaction.

To generate pME Lifeact mEGFP_3xHA (JDW 1322), a geneblock containing a consensus Kozak followed by the 17 amino acid F-actin binding element from Lifeact, then a flexible Gly-Gly-Gly-Ser linker, then enhanced GFP with monomerizing A206K mutation (mEGFP) (Zacharias et al., 2002), a Gly-Gly-Ala-Gly-Gly flexible linker, then a 3x HA epitope tag and a double stop codon was synthesized (Twist Biosciences) and then BP recombined into pDONR221.

pME Vhh-sfGFP-P2A-HA-iRFP-caax (JDW 1216) was synthesized by GeneUniversal and contains an actin nanobody derived from Addgene #159595 (an actin nanobody with a LOV domain insertion) (Gil et al., 2020). In this clone, the LOV domain was removed from the Vhh nanobody, and the open reading frame of Actin Vhh is followed by a flexible Arg-Ser-Leu-$(Gly_4Ser)_4$ linker and sfGFP (Gil et al., 2020) followed by an Ala-Ser-Gly-Ser-Gly linker, a Gly-Ser-Gly-P2A (GSG-P2A) cleavage peptide, a single HA epitope and then iRFP, followed by a linker with a NotI and XhoI site for insertion of another copy of iRFP to subsequently generate tdRFP, then a GHGTGSTGSGSSGRSG amino acid linker and the CAAX domain from HRAS for cell membrane localization. We note that this clone (like the parental template at Addgene) lacks three amino acids residues (Phe Val Lys) in the CD2 framework region (near amino acid 63). This did not appear to affect recognition of F-actin. This difference was corrected in subsequent actin nanobodies that were created as part of the MAGIC toolkit. We further modified this vector to insert another copy of iRFP, creating pME-Vhh-sfGFP-P2A-HA-tdiRFP-caax (JDW 1311).

pME-Actin Vhh-sfGFP (JDW 1215) contains a BamHI site, a BglI site, a consensus Kozak sequence, then an actin nanobody (VHH) followed by a HindIII site, and a flexible Gly-Gly-Gly-Gly-$Ser_{(x4)}$ linker, a KpnI site, then a single copy of super folder GFP (sfGFP), amplified from JDW 1216. While the original template lacks three amino acids (Phe-Val-Lys) in the CD2 framework around amino acid 63, this deficiency has been corrected in JDW 1215.

To generate pME Actin Vhh Halo Tag (JDW 1223), the "attB1-Actin-Vhh-Linker" insert was amplified from JDW 1215 (pME-ActinVhh-sfGFP) and then combined with an 831 bp HaloTag by PCR sewing. The final PCR product was amplified using oJDW 2100 (attB1 AgeI BamHI kozak homology), 5' GGGGACAAGTTTGTACAAAAAAGCAGGCaaccggtggatccAGCCACCATGGCTCAGGTGCAGCTGGTGG and oJDW 2101 (Homology 2x stop MluI SpeI attB2), 5'-CCCCACCACTTTGTACAAGAAAGCTGGGcACTAGTACGCGTttactaGCCGGAAATCTCGAGCGTCGAC. In the final pME clone, the Actin VHH nanobody is preceded by an AgeI, BamHI, then NcoI site, then a consensus Kozak sequence, a Gly-Gly-Gly-Gly-$Ser_{(x4)}$ linker

Development • Supplementary information

flanked 5' by a HindIII site and 3' by an XbaI site, then an 831 bp Halo Tag. Once amplified and then PCR purified, this was BP recombined with JDW 215 (pDONR-221).

pME Actin Vhh mCherry (JDW 1240) contains an AgeI, BamHI, and BglI site, then a consensus Kozak sequence, followed by an Actin nanobody (VHH) with a flexible linker (RSLGGGGSGGGGSGGGGSGGGG) flanked 5’ with HindIII and 3’ with KpnI, then mCherry followed by an XbaI site, 2 stop codons, then a SpeI site, all in an attL1/L2 flanked pME / middle entry backbone. The insert was created by PCR sewing using JDW 1215 (pME-Actin-Vhh-sfGFP) and JDW 1232 (pME-LSL-3xNLS-mCherry), and then recombined into pDONR221.

To generate pME Actin Vhh mNeonGreen-HA-pA (JDW 1248), which contains a Kozak then a VHH actin nanobody fused to an in frame linker (RSLGGGGSGGGGSGGGGSGGGGS) flanked 5’ by HindIII and 3’ by KpnI, followed by mNeonGreen, then an ASGSGGSG linker and a HA epitope and 2x STOP codon followed by an SV40 polyA then SnaBI and SpeI, the entire insert was amplified by PCR insert using JDW 1225 (p3E-Vhh-actin-mNeonGreen-HA) as a template and oJDW 2100, 5'-GGGGACAAGTTTGTACAAAAAAGCAGGCaaccggtggatccAGCCACCATGGCTCAGGTGCAGCTGGTGG and oJDW 2146, 5'-CCCCACCACTTTGTACAAGAAAGCTGGGcGGACTAGTTACGTAacgcgttaagatacat. The insert was then recombined into pDONR 221 (JDW 215) by a BP reaction.

pME-mScarlet3-Giantin (JDW 1356) was designed using the giantin DNA coding sequence from FRB-ECFP(W66A)-Giantin (Addgene #67903)(van Unen et al., 2015) and pmScarlet3-GiantinC1 (Addgene #189773)(Gadella et al., 2023) as templates to design a geneblock (Twist Biosciences) with a 5’ attB1 site, BamHI, SpeI, NcoI, then a consensus Kozak and a V5 epitope tag, followed by KpnI, then a flexible GSGGSGAS linker, followed by mScarlet3, then EcoRI, a (Gly-Gly-Gly-Gly-Ser)$_{x4}$ linker, HindIII, and a 376 bp Giantin fusion, stop codon, AscI-SalI-AccI-SnaBI-XbaI and an attB2 site. This insert was BP recombined into pDONR221.

pME-StayGold(c4)-Giantin (JDW 1355) was generated using a similar strategy, where a geneblock containing a 5’ attB1 site, BamHI, SpeI, NcoI, then a consensus Kozak and a V5 epitope tag, followed by KpnI, then a flexible GSGGSGAS linker, followed by oxStayGold(c4), then EcoRI, a (Gly-Gly-Gly-Gly-Ser)$_{x4}$ linker, HindIII, and a 376 bp Giantin fusion, stop codon, AscI-SalI-AccI-SnaBI-XbaI and an attB2 site was synthesized (Twist Biosciences) and BP recombined into pDONR221.

pME-mCerulean3-TUBB1A (JDW 1508) was generated by amplifying a fusion of mCerulean3 to the entire ORF (1353 bp) of human tubulin alpha 1b (TUBA) (minus the start codon/ATG of TUBA) from using oJDW 2506, 5’-GGGGACCACTTTGTACAAGAAAGCTGGGTgcaAAGCTTGCGGCCGCACTAGTcgttaagata

Development • Supplementary information

cattgatgagttt and oJDW 2507, 5’-GGGGACAAGTTTGTACAAAAAAGCAGGCTgcaGGTACCTTAATTAACTCGAGgcacgccaccATGGTGAG and mCerulean3-Tubulin-C-18 (Addgene # 55450) as PCR tmeplate. This attB1/B2 flanked amplicon was then inserted via BP reaction into pDONR221.

pME-FastFUCCI (JDW 1488) was created by amplifying the FastFucci cassette from pBOB-EF1a-FastFucci-Puro (Addgene #86849) (Koh et al., 2017) using oJDW 2415, 5'-GGGGACAAGTTTGTACAAAAAAGCAGGCTggtCTGACTGGTACCtcgtgattaattaagaattgggatccatgg and oJDW 2416, 5’-GGGGACCACTTTGTACAAGAAAGCTGGGTTGACTGGCGGCCGCTCAGTCAGATCTctacagggccttccgccg. Briefly, this contains monomeric Kusabira-Orange 2 fluorescent protein (mKO2) fused to human CDT1 (amino acid 30-120) as a G1 phase reporter and monomeric Azami Green (mAG) fused to human Geminin (amino acid 1-110) as a S-G2-M phase reporter. The resulting PCR amplicon was inserted into JDW 215 (pDONR221) via a BP reaction.

To generate pME-FastFUCCI-P2A-H2A-tdiRFP (JDW 1399), the mammalian FastFUCCI cassette (mKO2-hCdt1-P2A-mAG-hGem) from pBOB-EF1a-FastFucci-Puro (Addgene #86849) (Koh et al., 2017) was PCR amplified using oJDW 2369, 5’-ACAAAAAAGCAGGCTCCTTAGATTAATTAAGAATTGGGATCCATGG and oJDW 2370, 5’-CGTGGCGCCGCTGCCAGATCACAGGGCCTTCCGCCG. This FastFucci insert was cloned via Cold Fusion (System Biosciences, MC010B) into pME-Golgi-BFP-P2A-H2A-tdiRFP (JDW 1079) digested with XhoI - NotI, replacing the Golgi-BFP cassette.

pME-zFUCCI (JDW 1464) contains 3xFLAG-mCerulean-fused to amino acids 1-100 of zebrafish Geminin (zGem(1-100)) (S-G2-M reporter) followed by a P2A cleavage peptide and then mCherry fused to amino acids 1-190 of zebrafish Cdt1 (zCdt1(1-190)) (G1 reporter). zFUCCI was amplified by PCR using pTol2-UAS-Fucci (a kind gift from Dr. Karen Yaniv, Weizmann Institute of Science)(Jerafi-Vider et al., 2021) as a template and oJDW 2419 5’-GGGGACAAGTTTGTACAAAAAAGCAGGCTACTGTCAAGCTTCAGTCAGGATCCGGTATGGACTACAAAGACCATGACGG and oJDW 2420 5’-GGGGACCACTTTGTACAAGAAAGCTGGGTAGTCTGATCGATGACTCAGGTACCGGTttaCTCTTTGCGGGCAGTTTGTTG. The resulting PCR amplicon was cloned via BP reaction into pDONR221.

pME-zFUCCI-P2A-H2A-tdiRFP (JDW 1380) was created by amplifying zFUCCI using oJDW 2367 5’- ACAAAAAAGCAGGCTCCTTAGCCGGGATCCCTTCGATTG and oJDW 2368, 5’- CGTGGCGCCGCTGCCAGATCGCTCTTTGCGGGCAGTTTGTTGAG. The zFUCCI insert was cloned via Cold Fusion (System Biosciences, MC010B) into pME-Golgi-

Development • Supplementary information

BFP-P2A-H2A-tdiRFP (JDW 1079) digested with PacI and BglII, replacing the Golgi-BFP cassette to generate a tri-color reporter to indicate changes in cell cycle with red and green fluorescence and to label all cells with tandem dimer iRFP (tdiRFP).

In the LexA/LexO system, a ligand-dependent fusion between the DNA-binding domain (DBD) of the bacterial LexA repressor (residues 1-87) (Horii et al., 1981, Miki et al., 1981) and a truncated ligand-binding domain (LBD) of the human progesterone receptor (residues 640-914) is joined to the activation domain of the human NF-κB/p65 protein (residues 283-551) (Burcin et al., 1999). pME-LexA-mODC (JDW 937) was synthesized by Twist Biosciences and contains an optimized Kozak sequence followed by a nuclear localization sequence, then the bacterial LexA repressor (residues 1-87) (Horii et al., 1981, Miki et al., 1981) fused to a truncated ligand-binding domain (LBD) of the human progesterone receptor (residues 640- 914), joined to the activation domain of the human NF-κB/p65 protein (residues 283-551) (Burcin et al., 1999). This chimeric transcriptional regulator is fused to amino acids 422-461 of the PEST degradation domain of murine ornithine decarboxylase (mODC) to promote rapid protein turnover (Li et al., 1998), which should allow more a more responsive off rate when using the LexA/LexO system to drive targets.

pME-lexA-mODC-LexOP-35SCaMV (JDW 1348) was created by digesting pME-LexA-mODC (JDW 937) with NotI and NheI and inserting a SV40-polyA cassette followed by a terminator sequence from the nopaline synthase (NOS) gene in *Agrobacterium tumefaciens*,

and four ColE1 operator sites, followed by the minimal Cauliflower mosaic virus 35S (CaMV 35S) promoter (collectively termed the LexA Operon) to create an entire LexA/LexO cassette in a single middle entry clone.

pME-LexA-mODC-LexOP-c-fos (JDW 1359) was created by digesting pME-lexA-mODC (JDW 937) with NheI and NotI. The SV40 polyA-RNAP II transcriptional pause signal-6x ColE1 Operator-minimal murine *c-Fos* promoter cassette from JDW 1254 (p5E-lexAOP-cFos) was amplified by PCR using oJDW 2343 5'-TGATCCTCCACGTAgcggccaacttgtttattgcagcttataatggttacaaa and oJDW 2344 5'-AATTAATCTAGAGTTGCTAGagttgctcgctgcagtcg and inserted into the gel purified backbone via Cold Fusion.

To generate pME-TAEL2.0-5xC120-minpro (JDW 1109), an insert containing an attB1 and attB2 flanked insert containing the T4A EL222 (light activatable transcriptional regulator, aka TAEL 2.0) (LaBelle et al., 2021) with a carboxy terminal NLS followed by an SV40 polyA then a transcriptional pause site and a NOS terminator sequence to prevent transcriptional readthrough, then a 5x C120 repeat DNA binding sequence followed by the

Development • Supplementary information

murine *c-Fos* minimal promoter, flanked with a NdeI to HindIII site external to the attB sites (and a BamHI and PacI site at the 5’ side and SpeI and XbaI site at the 3’ side interior to the attB sites) was synthesized by Gene Universal and recombined via BP reaction into pDONR221.

pME TAEL2.0 (JDW 1324), which contains an attL1/L2 flanked TAEL2.0 cDNA followed by an SV40 late polyA, a transcriptional pause site, then a NOS terminator, was created by BP recombination of pDONR221 with an attB1/attB2 flanked gene block containing the insert described above.

pME-LiCre (JDW 1119) contains a chimeric fusion between the photropin 1 LOV2 domain of *Avena sativa* (AsLOV2) fused to the αA-terminal helix of a destabilized Cre recombinase carrying mutations in its N- and C-terminal domains (p.E340A, p.D341A) engineered by the Yvert lab for light-induced dimerization (Duplus-Bottin et al., 2021). An attB1/B2 flanked AsLOVE2-Cre^{E340A,D341A} insert was amplified by PCR using pGY577 (Addgene #166663) (a gift of Gaël Yvert) (Duplus-Bottin et al., 2021) as a template and the following oligos: oJDW 1950 (Kozak + SalI) 5’-aaaaGTCGACgccaccatgggtccaaaaaagaagagaaaggtagatcc, and oJDW 1951 (HindIII) 5’-atccagaggttgattggatccaagc. The amplicon was then digested and inserted into JDW 457 (pME-MCS) digested with the same enzymes. Of note, we found Addgene pGY577 #166663 contains an in frame 24 bp deletion of one of the 3 NLS sequences, so only 2 remain in the plasmid (and in JDW 1119).

pME-3xNLS-$(G_2SG_2)_2$-Cre (JDW 1149) encodes a 3xNLS-Cre, where the insert is flanked 5' by XhoI and XbaI before the Kozak, then Bgl-II, SacI, PstI, and SalI after the 3x NLS (in the Gly-Gly-Ala-Gly-Gly linker) and 3' by SpeI. The 3x-NLS-Cre insert with flanking attB1/B2 sites was amplified by PCR from JDW 518 (pUCIDT-3xNLS-Cre_KAN) (synthesized by IDT) using the following oligos: oJDW 2008 (pME 3xNLS-Cre FWD) 5’-GGGGACAAGTTTGT ACAAAAAAGCAGGCTCTCGAGTTATTCTAGACGCCACC, and oJDW 2009 (pME 3xNLS-Cre REV), 5’-CCCCACCACTTTGTACAAGAAAGCTGGGACTAGTCTACTACTAATCGCCATCTT CC.

pME-se-iCreI-HA (JDW 1203) contains an HA-tagged, self-excising, improved Cre (iCre). This mammalian codon optimized iCre contains fewer CpG islands, no 5’ UTR, a strong Kozak, an amino-terminal NLS from SV40 (Shimshek et al., 2002). Further, the open reading frame of Cre contains a functional intron (a chimera between introns from the human β-globin and immunoglobulin heavy chain genes) to prevent basal expression in prokaryotes (Kaczmarczyk and Green, 2001). The entire cassette is flanked by two *loxP* sites in a pUC57 Kan backbone with flanking *attL1* and *attL2* recombination sites. The entire *loxP* flanked insert can be removed via digesting with PacI or AgeI at the 5' end and EcoRV or MluI at the 3' end. If a polyA needs to be inserted downstream of Cre, it can be inserted between the

Development • Supplementary information

NotI and ClaI sites, or downstream at XbaI. HindIII digestion will remove the entire Cre insert, but retains the flanking loxP sites. The HA tag can be removed via BamHI. The 5' region downstream of the loxP site can be cut with KpnI, EcoRI, or NcoI (downstream of the Kozak) to remove the insert.

pME-3xNLS-mTagBFP2-2xHA-Cre (JDW 918) is a middle entry containing Cre recombinase c-terminally fused directly to a nuclear localized mTagBFP2 blue fluorescent reporter with an intervening 2x HA tag that was PCR amplified from pCS2-3xNLS-mTagBFP2-2x-HA-Cre (to be described elsewhere) using the following oligos: oJDW 1346 (attB1-kozak-nlsTagBFP-FWD) 5’-aaaaggggACAAGTTTGTACAA AAAAGCAGGCT*cgccaccATG*CCAAAAAAGAAG and oJDW 1347 (attB2-*stop*-Cre-Rev) 5’-aag gggACCACTTTGTACAAGAAAGCTGGGT*cta*ATCGCCA TCTTCCAGCAGG. Briefly, the insert contains a consensus Kozak, followed by a 3xNLS (SV40), then a flexible linker (Gly-Ala-Gly-Gly-Arg), a BglII site, then mTagBFP2, a c-terminal HindIII site, three Gly residues, 2 HA epitope tags, a SalI site, a flexible linker (Gly-Arg-Arg-Gly-Pro-Gly-Gly-Ala-Gly-Gly), then Cre, and a stop codon. The resultant PCR amplicon was purified, then recombined in a BP reaction with pDONR221.

To create pME-nls-EGFP-Cre (JDW 1329), a geneblock containing an attB1 site, PacI and SpeI sites, then a Kozak consensus sequence, a single NLS from SV40, then a flexible Gly-Gly-Ala-Gly-Gly (G_2AG_2) linker fused to EGFP, followed by a SGTALDGESSGSGSESDS linker, an NcoI site, the open reading frame of Cre recombinase, followed by three stop codons and an NheI site at the 3' end before the attB2 site was BP recombined into pDONR221.

To generate pME-mTurquoise-GlySerGly(GSG)-P2A-iCre (JDW 920), mTurquoise was amplified from pmTurquoise-H2A (Addgene #36207)(Goedhart et al., 2012) then cloned into pAAV-EF1a-EGFP-P2A-iCre to replace EGFP via BamHI to BsrGI digestion. The resulting pAAV-EF1a-mTurquoise-P2A-iCre vector was used as a template for PCR with the following oligos: oJDW 1344 (attB1_mTurquoise-Fwd): 5’-aaggggACAAGTTTGTACAAAAAAGCAGGCT *gccaccATGG*TGAGCAAGG and oJDW 1345 (attB2-stop-iCre-Rev) 5’-aaggggACCACTT TGTACAAGAAAGCTGGGT*tta*GTCCCCATCCTCGAGCAG. The resultant PCR amplicon was purified, then recombined in a BP reaction with pDONR221.

To generate pME-EGFP-GSG-P2A-iCre (JDW 921), pCS2-EGFP-GSG-P2A-iCre (to be described elsewhere) was used as a template for PCR with the following oligos: oJDW 1340 (attB1-*kozak*-EGFP-Fwd): 5’-aaggggACAAGTTTGTACAAAAAAGCAGGCT*gccaccATGG*TGAG CAAGGGCG AG and oJDW 1341 (attB2-stop-iCre-Rev): 5’-aaggggACCACTTTGTACAAGAAA

Development • Supplementary information

GCTGGGT*tta*GTCCCCATCCTCGAGCAGC. The resultant PCR amplicon was purified, then recombined in a BP reaction with pDONR221.

pME-mScarlet-P2A-iCre (JDW 917) was created by PCR of an existing template (pCS2-mScarlet-P2A-iCre) with the following primers: oJDW 1342 (attB1-*kozak*-mScarlet-Fwd): aaggggACAAGTTTGTACAAAAAAGCAGGCT*gccaccATGG*TGAGCAAGG, oJDW 1343 (attB2-*stop*-iCre-Rev): 5'-aaggggACCACTTTGTACAAGAAAGCTGGGT *tta*GTCCCCATCCTCGAGCAG. The resultant amplicon was PCR purified, then recombined in a BP reaction with pDONR221.

pME-*loxP*-3xSTOP-*loxP* (JDW 869) was created by removing H2B-mCerulean from pME-*loxP*-H2B-mCerulean-STOP-*loxP* (a gift of Drs. Ching-Ling Lien and Michael Harrison, USC) by digestion with HindIII and NotI, then cloning in a gene block with a bGH and SV40 polyA sequence upstream of the already present SV40 polyA to generate pME-HindIII-BamHI-SV40pA-bgHpA-SV40pA-KpnI (JDW 869).

pME-lox-3xNLS-mCherry-V5-SV40pA-stop-lox (JDW 1232) was created by digesting pME-Lox-3xStop-Lox (JDW 869) with 5' HindIII and 3' XhoI to remove the first SV40 pA, and then 3xNLS-mCherry-V5-Sv40pA was amplified by PCR using an existing template in the lab and oJDW 2061, 5'-TTATACGAAGTTATcggagcaagcttCATATGGTCGACGATATCATC GATGCTGC and oJDW 2062, 5'-GGTATGGCTGATTATGATCCCTCGAGacgcgttaagatacattgat gagtttggacaaacc and cloned into the backbone via cold fusion cloning.

pME-Dre-nls (JDW 31) was amplified from pCAGGS-Dre-nls (a gift from A. Francis Stewart, Biotechnology Center TU, Dresden, Germany) using the following oligos: FWD (Topo-Kozak-Dre F) 5'-CACCGTCGCCACCATGGGT GCTAGCGAGCTGATCATC and REV (DRE-NLS-Rev-double stop) 5'-TCATCACACTTTCCTC TTCTTCTTAGGACC. The resultant PCR amplicon was purified and directionally TOPO cloned into pENTR/D-TOPO. pME-Dre-ERT2 (JDW 1344) was made by PCR stitching Dre, minus the nls, to ERT2 (Feil et al., 1997), and inserting a CVRGS linker (5'-TGCGTACGCGGATCC-3') (Logie et al., 1998) between Dre and ERT2.

To generate a rox-flanked red fluorescent reporter for use with Dre recombinase, a PacI-AgeI-*rox*-HindIII-KpnI-SacII-Kozak-NcoI-3xNLS-BamHI-mCherry-V5-SnaBI-NotI-ClaI-2xSV40 late polyA-HindIII-PstI-*rox*-XhoI-EcoRV-MluI insert was cloned between attL1 and attL2 in pUC57 to generate pME-*rox*-mCherry 2x-stop-*rox* (JDW 1167).

3xFLAG-human Ubiquitin was amplified by PCR using pCDNA3-3xFlag-Ubiqutin (a gift of Dr. Andre Catic at Baylor College of Medicine) and the primers FWD 5'-GGGGACAAGTTTGTACAAAAAAGCAGGCTACTAGTGCTAGCGCCACCACCaccatggactac aaagaccatgacgg and REV 5'-GGGGACCACTTTGTACAAGAAAGCTGGGTggatcctctagagtcgac tggtacc. The purified PCR

Development • Supplementary information

product was used in a BP reaction with pDONR221 to generate pME-3xFlag-hUbiquitin (JDW 1098).

pME-myc-BirA (JDW 1210) was generated by synthesizing a gene blocking containing attB1-attB2 sites flanking an insert containing a Kozak sequence followed by a MYC epitope tag and the promiscuous R118G mutant of the *E. Coli* protein biotin ligase (Cronan, 2005) and then a stop codon.

A second variant, pME-Myc-BioID2 (JDW 1095), which contains a promiscuous R40G mutant of a biotin protein ligase from *Aquifex aeolicus* (known as BioID2), was created by PCR amplification of myc-BioID2-MCS (Addgene 74223, a kind gift of Dr. Kyle Roux)(Kim et al., 2016a) with oJDW 1856 (AttB1_NheI_SalI_ClaI_Kozak_Myc_BIOID2) 5'-GGGGACAAGTTTGTACAAAAAAGCAGGCTAAGTCGACATCGATgccaccatggaacaaaaactcatctc and oJDW 1853 (BioID2_2STOP_KpnI_HindIII_AttB2_REV) 5'-GGGGACCACTTTGTACAAGAAAGCTGGGaTTAATTaaaagcttGGTACCTTATTAgcttcttctcaggctgaactcgcc. The resulting PCR product was BP recombined into pDONR221.

A second BioID2 variant for creating fusion proteins was created by inserting an AgeI (Thr-Gly) site followed by a flexible linker (Gly-Ser-Gly-Gly-Ser-Ala-Arg-Arg-Gly Ala-Ala-Ala-Ala-Ser) that includes AscI and NotI sites, followed by a stop codon, and XhoI, KpnI, BglII, SnaBI, and XbaI into pME-BioID2 to create pME-BioID2-MCS (JDW 1298).

pME 3xHA-BirA-T2A-mCherry-caax (JDW 889) was generated by PCR using the following primers oJDW1306 (attB1_*kozak*_HA_BirA FWD): 5'-aaggggACAAGTTTGTACAAAAAAGCAGGCT*gcc acc*ATGGCCACCTATG, oJDW1307 (attB2r_*stop*_CAAX REV): 5'-aaggggACCACTTTGTACAA GAAAGCTGGGT*tca*GGAGAGCACACACTTGCA, with Addgene #80056 (Trinh et al., 2017) as a DNA template.

pME rtTA3G (JDW 1230) was created by performing PCR using an existing template (p3E-C-EFS-rtTA3G, JDW 1214) and the following primers: oJDW 2124 (attB1 rtTA3G FWD) 5'-GgggACAAGTTTGTACAAAAAAGCAGGCTATGTCTAGGCTGGACAAGAGCAAAG and oJDW 2125 (rtTA3 3' attB2 REV) 5'-ggggCCACTTTGTACAAGAAAGCTGAATTCCTATCATTACCCGGGGAGC. The resulting PCR product was PCR purified, then BP recombined with pDONR221 and create this middle entry plasmid.

Hyperactive PBase (Yusa et al., 2011) (a gift of Dr. Benjamin Arenkiel at Baylor College of Medicine) and a downstream WPRE (without a polyA) were subcloned from Cdh5-hyPBase-WPRE-pA (to be described later) into pME between EcoRI and SalI to generate a pME-hyPBase-WPRE (JDW 679). Note, that due to MTA issues with the patent

holder for *piggyBac* technology, Hera BioLabs, this construct cannot be distributed by Addgene.

pME-EGFP-miR-MCS (JDW 1231) was based on pg4a (a kind gift of Dr. Michael McManus at UCSF). Briefly, using pEGFP-N3 (Clontech) as template, an artificial MCS and intron from the human *HPRT* locus were inserted into EGFP between Valine 164 and Lysine 165 of EGFP. Multiple cloning sites were included for subsequent insertion of genomic loci containing microRNAs. The empty intron containing GFP insert was then amplified with flanking attB1 and attB2 sites to create pME-EGFP-miR. For cloning microRNAs into pME-EGFP-miR (JDW 1231), oligos including approximately 500-700 bp of sequence surrounding either the *miR-500* or *miR-126* locus were designed for insertion into the BamHI and PstI sites to create pME-EGFP-miR-500 (JDW 8) and pME-miR-126 (JDW 1401).

To generate pME-β-Catenin^{S33Y} (JDW 419), a dominant active human β-Catenin (β-Catenin^{S33Y}) cDNA insert was amplified from pCDNA-β-Catenin^{S33Y} (Addgene #19286) (Kolligs et al., 1999) using the following primers: FWD (Attb1 KOZAK S33Y FWD) 5’-GGG GAC AAGTTT GTA CAA AAA AGC AGG CT G GAT CCG CCA CCATGG CTA CTC AAG CTG ATT TGATGG and REV (Attb2 FLAG REV) 5’-CCC CAC CACTTT GTA CAA GAA AGCTGG GGC GGC CGCTTA CTT GTC ATC GTC. The resultant PCR product was recombined by BP reaction into pDONR221.

pME-V5-mClover3-KRAS4A-WT (JDW 812) was created by cutting pAAV-sCAG-mClover3-KRAS4A-WT with SpeI and AscI to shuttle mClover-KRAS into an existing pME clone using the same sites. A similar strategy was used to generate pME-V5-mClover3-KRAS4A-G12D (JDW 813), as well as pME-V5-mScarlet-I-KRAS4A-WT (JDW 822), pME-V5-mScarlet-I-KRAS4A-G12V (JDW 823), pME-V5-mTagBFP2 (JDW 830), pME-V5-mTagBFP2-KRAS4A-WT (JDW 831), and pME-V5-mTagBFP2-KRAS4A-G12D (JDW 832).

pME-EGFP-MYC (JDW 1441) was created by amplifying EGFP-Myc from p3E-IRES-EGFP-Myc-SV40-pA (JDW 1438) using oJDW 2428 5'-CAAGTTTGTACAAAAAAGCAGGCTTCAGTGACTAGTtaGGTACCtaTTAATTAAggccgATGGTGAGCAAGGGCGAG and oJDW 2429 5'-GGGGACCACTTTGTACAAGAAAGCTGGGTC TGACTGCGGCCGCtagttattacgcacaagagttccgtagc and recombining the resulting PCR product into pDONR221 via a BP reaction.

p3E Construct Generation:

Development • Supplementary information

Unless stated otherwise, 3’ ENTRY vectors were produced by PCR amplification of the desired template using attB2/B3r-flanked primers, or direct synthesis of the fragment with flanking attB2/B3 sites, followed by a BP reaction with pDONR P2R-P3 (Invitrogen). In some cases, we used a modified p3E ENTRY vector that contains a multiple cloning site flanked by the P2R and P3 sites, known as p3E-MCS (Addgene #75174)(Don et al., 2017), for restriction enzyme-based cloning.

p3E-WPRE-SV40-pA (JDW 922) was generated by PCR amplification of the WPRE and SV40 poly(A) signal cassette from pTol2-E1b-EGFP-WPRE-DV (JDW 576) using the following primers: oJDW 1378 5’-ACCCAGCTTTCTTGTACAAAGTGGTCAACCTCTGGATTACAAAA and oJDW 1379 5’-GGGGACAACTTTGTATAATAAAGTTGAACTTGTTTATTGCAGCTTA. The PCR product was then BP recombined into pDONR P2R-P3.

p3E-WPRE-bGH-pA (JDW 1221) was generated by PCR amplification of the WPRE and bovine growth hormone poly(A) signal cassette from pAAV-EF1a-FLEX-lifeact-mScarlet-HA-WPRE-bGHpA (JDW 719) using the following primers: oJDW 2139 (Fwd WPREBgH) 5'-ggggACCCAGCTTTCTTGTACAAAGTGGActatcgataatcaacctctggattacaa and oJDW 2140 (rev bghwpre) 5'-ggggCAACTTTGTATAATAAAGTTGGGTACCgatgcaatttcctcattttattagg. The PCR product was then recombined into pDONR P2R-P3.

p3E-mCherry-SV40-pA (JDW 1417) was created by ordering a gene block (Twist Biosciences) with mCherry followed by an SV40 pA, all flanked by attB2/attB3 sites. This insert was then BP recombined with pDONR P2R-P3.

p3E-EF1a-pA (JDW 1222) was created by amplifying the rat EF1-poly(A) using the following primers: oJDW 2137 (EF1a fwd) 5'-ggggACCCAGCTTTCTTGTACAAAGTGGAACGCGTATTATCCCTAATACCTGCCACC and oJDW 2138 (3' EF1Pa rev) 5'-ggggCAACTTTGTATAATAAAGTTGGGTACCAGCTTTCTATGCAACCCAAG and pWhere-H2B-EGFP-Dest (JDW 410) as a template, then the product BP recombined with pDONR P2R-P3.

p3E-H2A-mCherry_SV40pA (JDW 967) was created by amplifying H2A-mCherry from JDW 714 (pME_V5_KRAS_G12V_p2a_H2AZ/F_mCherry) with the following primers: oJDW 1529 (5’-aaaaGGATCCTAGGATGGCAGGTGGAAAAGCAGG) oJDW 1018 (CTTTGTACAAGAAAGCTGGGAGATCTCTCGAGCTATCATTACTTGTACAGCTCGTCCATGC) to add BamHI and XhoI restriction enzyme sites. The PCR product and vector JDW 817 (p3E-V5-mScarletI-KRAS4A-G12V-SV40pA) were digested with BamHI and XhoI to remove the insert in the backbone, and then ligated together and then transformed into Stbl3 competent cells.

Development • Supplementary information

A 3' entry clone for three-way multisite gateway LR reactions, containing a V5-mScarlet-I fluorescent reporter with a stop codon followed by a poly(A) signal, p3E-V5-mScarlet-I-SV40pA (JDW 968), was created by amplifying the V5-mScarlet insert from JDW 807 (attB-AC-zsY-V5-mScarlet-I-KRAS4AG12V) with oJDW 1037 (5'-ATATCTCTCGAGGGCGCGCCTTACTTGTACAGCTCGTCCATGCC) and oJDW 1230 (5'-agaattGGATCCACTAGTGCTAGCgccaccatgGGTAAGCCTATCCC) to add BamHI and XhoI cut sites. The insert and JDW 817 (p3E-V5-mScarletI-KRAS4A-G12V-SV40pA) were digested with BamHI and XhoI to remove the insert in the backbone, and then ligated together and then transformed into Stbl3 competent cells.

p3E-V5-mClover3-SV40-pA (JDW 1318) was constructed by performing a BP reaction with a gene block sequence containing attB2-V5-mClover3-SV40-pA-attB3 and pDONR P2R P3.

To generate p3E-V5-mTagBFP2-SV40-pA (JDW 871), JDW 824 (pAAV-sCAG-V5-mTagBFP2-WPRE-SV40pA) was cut with SpeI and AscI and the resulting V5-mTagBFP2-SV40-poly(A) fragment was then inserted into JDW 814 (p3E-V5-mClover::KRAS4A(WT)-SV40pA) that was cut and gutted with the same enzymes. V5 is separated from the mTagBFP2 insert by a flexible Gly-Gly-Ala-Gly-Gly (G_2AG_2) linker.

To generate p3E-mCherry-T2A-HRAS-G12V-SV40-pA (JDW 1188) a gene block was synthesized with attB2 and attB3 sites flanking an mCherry followed by a T2A peptide cleavage sequence and then human HRAS G12V and an SV40 pA. The insert was then BP recombined with pDONR P2R-P3.

To generate p3E-Vhh-Actin-mNeonGreen-HA-SV40-pA (JDW 1225), a gene block was made with attB2 attB3 sites flanking a Kozak and a VHH actin nanobody fused to an in frame linker (5' HIII to 3' KpnI) (RSLGGGGSGGGGSGGGGSGGGGS) followed by mNeonGreen then a ASGSGGSG linker and a HA epitope tag and 2x STOP codons followed by an SV40 polyA then SnaBI and SpeI. The insert was then BP recombined with pDONR P2R-P3.

To generate p3E-IRES-V5-(n2)oxStayGold(c4)-pA (JDW 1364), a geneblock containing attB2-IRES-V5-$GGGGS_{(x3)}$-(n2)oxStayGold(c4)-SV40-pA-attB3 was BP recombined with pDONR P2R-P3.

p3E-IRES-3xFLAG-3xNLS-mScarlet-I3-SV40-pA (JDW 1360) was created by BP recombination of a geneblock containing attB2-IRES-3xFLAG-3xNLS-mScarlet-I3-SV40-pA-attb3 with pDONR P2R-P3.

p3E-IRES-3xFLAG-3xNLS-mTagBFP2-SV40-pA (JDW 1361) was created by BP recombination of a geneblock containing attB2-IRES-3xFLAG-3xNLS-mTagBFP2-SV40-pA-attb3 with pDONR P2R-P3.

Development • Supplementary information

p3E-IRES-H2A.Z-mCherry-SV40-pA (JDW 1256) was created by BP recombination of a geneblock containing attB2-IRES-H2A.Z(zebrafish)-mCherry-SV40-pA-attb3 with pDONR P2R-P3.

p3E-EFS-rtTA/rtTA3 (JDW 1214) was created by ordering a gene block (Twist) with the human EFS/EF1a-core promoter upstream of an rtTA-3G (Tet-On, 3rd generation) transcriptional transactivator followed by an SV40 poly(A) signal, followed by recombination with pDONR P2R-P3.

p3E-IRES-Luciferase-WPRE-bGH-pA (JDW 1509) was created by ordering a gene block (Twist Biosciences) with the ECMV IRES followed by optimized firefly luciferase (Luc+/Luc2) open reading frame followed by a WPRE and bGH pA, all flanked by attB2/attB3 sites. This insert was then BP recombined with pDONR P2R-P3.

Destination Vector Generation:

The backbone for pCAGEN is pCAGGS (Niwa et al. Gene 108, 193-199 (1991)), from Dr. J. Miyazaki (Osaka University), and the multiple cloning site (MCS) of pCAGGS was modified by adding an EcoRI, XhoI, EcoRV and NotI site to generate pCAGEN. pCAGEN, a kind gift from Connie Cepko (Addgene Plasmid #11160)(Matsuda and Cepko, 2004), was further modified by inserting an *attR1-ccdB*-Cm^{R}-*attR2* Gateway cassette into the EcoRV site of MCS to generate pCAGEN-DEST. pCAGGS-DEST-WPRE variant was generated by insertion of WPRE bGH polyA fragment by Cold Fusion (SBI Systems) cloning between the XhoI and BglII sites.

For pCAGEN-Dest-IRES-myr-BFP (JDW 491), pCAGEN-Dest-IRES-myr-mKate2 (JDW 494), and pCAGEN-Dest-IRES-myr-EGFP (JDW 495), PCR amplicons of each respective fluorophore, including an additional 18 amino terminal amino acids from GAP43 (MLCCMRRTKQVEKNDEDQKI) (Denny, 2006, Heim and Griesbeck, 2004) that anchors proteins to the inner plasma membrane, were inserted by restriction digest and ligation into pCAGEN-Dest. Constructs were transformed into chemically competent *ccd*B- bacteria (strain, Invitrogen) and then plated on LB agar plates with ampicillin and chloramphenicol. Transformants were screened by colony PCR for the presence of the insert, then confirmed by Sanger sequencing.

The Mef2c-F6/frag3 plasmid (Dodou et al., 2004), a kind gift of Dr. Brian Black (UCSF), was digested with XhoI and SalI to isolate the 3,970 bp SHF/AHF enhancer fragment, while pCAGEN was cut with SpeI and XbaI to remove the CAGGS promoter. The resulting fragments were blunted and ligated together to make the Destination vector pMef2c-AHF-Dest (JDW 476).

Development • Supplementary information

The β-MHC-promoter (murine *MyH7*) clone #32 in a pBSKII backbone, a gift from Dr. Jeffrey Robbins' lab (Addgene #53963)(Rindt et al., 1993), was modified by digestion with HpaI and insertion of an Destination Reading Frame "A" (RFA) cassette to create the embryonic, pan-cardiomyocyte transgenic expression vector pβ-MHC-promoter Dest (JDW 26).

The African clawed frog *Xenopus laevis Myosin Light Chain 2* promoter (Latinkic et al., 2004), *XMLC2*-pro, is sufficient to drive expression of Cre recombinase in cardiomyocytes from early embryogenesis through adulthood in mice, with labelling evident in all four chambers and the cardiac outflow tract (Breckenridge et al., 2007). This 3.0 kb region was amplified by PCR using 5' and 3' oligos that both contained a HindIII site, and pEF-DEST51 (Invitrogen) was digested with the same enzyme to remove the EF1a promoter in exchange for *XMLC2-pro* to generate pxtMLC2-Dest (JDW 7).

pGlast-DEST (JDW 1009) was generated by removing PBase from Glast-PBase (a kind gift of Dr. Benjamin Deneen, Baylor College of Medicine) via PspXI and NcoI, then inserting an *attR1*-Cm^R-*ccd*B-*attR2* cassette downstream of the human *GLAST* promoter via Cold Fusion cloning. The Gateway cassette was amplified with the following primers: FWD 5'-cctcgttactgcttgatGGGCTCGAGATCc tgttttgacctccatagaagacaccg and REV 5'-CGGCCGGCCGCCCCGACTCTAGATGCATGCattc gatgggggatcccttcg using JDW 931 (pB_TetOn_DEST_EFS_mODC_rtTA_IRES_NEO) as a template.

pCAGGS-*rox*-nKateV5-*rox*-Dest-FRTNeo (JDW 472) was generated by cloning 3x-NLS-mKate2-V5 followed by a rabbit globin polyA sequence between *rox* recombination sites downstream of a CAGGS promoter. Then, a Gateway RFA Destination cassette and a rabbit globin polyA sequence were cloned downstream to generate pCAGGS-rox-nKateV5-rox-Dest. Subsequently, a SacI-SalI fragment containing an FRT-PGK-FRT cassette from pK11 (Courtesy of Dr. Gail Martin at UCSF) was inserted 3 to the Dest cassette in the opposite orientation to generate pCAGGS-rox-nKateV5-rox- FRT-murine PGK promoter-NEO-FRT. This substrate was used in an LR reaction with pME-myr-mTagBFP2-Flag (JDW 1183) to generate pCAGGS-rox-nKateV5-rox-myrBFPflag-frt-PGK-NEO-frt (JDW 1217).

pB-Tet-On-DEST-EFS-mODC-rtTA-IRES-NEO (JDW 931) (pB-Tet-On 3.0) was created by gutting pB-TA-ERN (Addgene # 80474), a gift from Knut Woltjen, with AvrII. Next, a fragment containing a transcriptional pause site followed by the human EF1α minimal core promoter (also known as EF1α short, or EFS) and destablized rtTA-3G (reverse tetracycline transactivator, 3rd generation) (synthesized by Twist Biosciences) was inserted to create a *piggyBac* transposon flanked "Tet-On" Destination vector that is compatible with pME / pDONR221 middle entry clones for dox/tet-inducible expression in mammalian cells.

Development • Supplementary information

pB-Tet-on-DEST-hEF1a-mODC-rtTA-IRES-NEO (JDW 1086) (pB-Tet-On 2.0) was created by digesting pB-Tet-On 2.0 (JDW 931) with SpeI and AsiSI and inserting the human EF1a promoter (amplified using the following primers) oJDW 1845 (Human EF-1a FWD + AsiSI) 5'-aaaaGCGATCGCTTCTAGGTCTTGAAAGGAGTGCCTCG and oJDW 1846 (Human EF-1a REV + SpeI) 5'-aaaaACTAGTTACCAAGCTAATTCCTCACGACACC and JDW 10 (pEF-miR-500-EGFP) as a template.

pB-Tet-Off-FLEX-Dest-EFS-mODC-tTA (JDW 936), a *piggyBac* ITR flanked, Cre-dependent, "Tet-Off" vector was created by amplifying an *attR2*- ccdB-Cm^R- *attR2* with the following primers oJDW 1398 (FWD) 5'-AAAAGCTAGCGATTCGAATTCAAGGATCAACAAGTTTGTAC and oJDW 1399 [REV] 5-AAAAGGCGCGCCGCTCGAGAGGATCAACCACTTTG, using pCS2-DEST as a template for the PCR reaction. The Destination cassette was inserted downstream of a TRE-tight promoter (composed of 7x, Tet operons and a minimal CMV promoter) via NheI and AscI restriction enzyme sites between two inverted pairs of flanking *lox2272* and *loxP* sites (double inverse oriented *loxP* and *lox2272* recombination sites, also known as DIO or FLEX), followed by a WPRE and bGH polyA sequence, another polyA signal, then an RNA Pol II transcriptional pause site from the human *α2 globin* gene, followed by a human EFS promoter and a destabilized tTA (mODC-tTA, also known as d2tTA) followed by an SV40 polyA signal, all flanked by two *piggyBac* ITRs.

pTol2-2xIns; *hsp70*-zCreI-BFP-mODC (JDW 972) was synthesized by Gene Universal. Briefly, a 5' Tol2 arm is adjacent to a *hsp70* (heat shock promoter) upstream of zCre, a zebrafish codon optimized Cre based on Cre.zf1 (Horstick et al., 2015) that contains an amino-terminal nuclear localization sequence from SV40 (PKKKRKV). The bacterial 5′ untranslated region was replaced with an optimized Kozak translational start site, and the amino and carboy-terminal halves of zCre are separated by an artificial intron (zCreI). Inclusion of the artificial intron limits bacteria's ability to synthesize Cre and thus limits recombination in prokaryotes, yet allows for robust recombination in eukaryotic cells (Kaczmarczyk and Green, 2001). The carboxy-terminus of zCreI is followed by a flexible linker (ASGGAGGAGST) that contains NheI and BamHI sites for further manipulation, followed by mTagBFP2 (Ile174>Ala) (mammalian codon optimized)(Subach et al., 2011) linked to a 37 amino acid destabilization peptide (the so called "PEST" domain) from murine ornithine decarboxylase (mODC)(Loetscher et al., 1991) to prevent accumulation of Cre protein in vivo. Downstream is an SV40 polyA, followed by 2 cHS4 globin insulator sequences, then another polyA before the 3' Tol2 arm.

pDestTol2-2xIns; *hsp70*-zCreI-BFP-mODC (JDW 1002) was created by digesting JDW 972 (pTol2-2xIns; *hsp70*-zCreI-BFP-mODC) with SalI and Cold Fusion (SBI Bioscience) was used to insert a Gateway *attR3-ccdb-chlor-attR4* recombination cassette

Development • Supplementary information

upstream of the polyA. The ccdb and chloramphenicol Gateway cassette from pTol2Dest-pA2 (Kwan et al., 2007) was amplified with the following primers: oJDW 1630 (FWD): 5'-AACACAGGCCAGATCCTAGGGGGCCCGTTTAAACGCCATG ATTACGCCAA GCTATCAACTTTGT and oJDW 1631 [REV]: 5'-ATTTGTAACCATTATAAGCT GCAATAAACAAGTTGATCATCATCGATGGTACCGTAAAACGAC. There is a frameshift mutation at the c-terminal end of the chloramphenicol sequence; but the mutation has been carried over from pTol2Dest-pA2 and does not affect chloramphenicol resistance.

pDestTol2-2xIns; *hsp70*-zCreI-BFP (JDW 1184) was created by digesting pDestTol2-2xIns; *hsp70*-zCreI-BFP-mODC (JDW 1002) with MluI and AsiSI to remove the entire zCre-intron-mTagBFP2-mODC cassette. A shorter cassette without the mODC tag was amplified using the primers oJDW 2064 (BFP-G2AG2-NLS-AsiS1) 5'-AATTACGCGTGCCGCA TATGGCCACCatggtgcccaagaagaagagg and oJDW 2065 (zCreI-Mlu1) 5'-AATTACGCGTGCC GCATATGGCCACCatggtgcccaagaagaagagg, and inserted using those same restriction sites.

pDestTol2-2xIns; *fli1ep*-zCreI-mTagBFP2 (JDW 1218) was created by digesting JDW 1184 (pDestTol2-2xIns; *hsp70*-zCreI-BFP) with MluI and SpeI to remove the *hsp70* promoter. Then the chimeric endothelial-enhancer/promoter from zebrafish *fli1ep* (Villefranc et al., 2007), amplified from JDW 897 (pTol2-fli1a-mScarlet; mClover3-*Dll4-F2*) using the following primers: oJDW 2090 (SpeI 5' homology JDW 897) 5'-ggccACTAGTATCTCATCTTGACCCATAAACATACACTAAAACC and oJDW 2091 (MluI 3' homology JDW 896) 5'-aaggccACGCGTCGGATGGTTTTTTTTCCTCTAAATTTGGGAA and inserted using MluI and SpeI to generate a Gateway compatible, multisite Destination plasmid with *fli1ep*-driving Cre and mTagBFP2 in the backbone.

LR Cloning:

Gateway two vector, three vector, and four vector recombination was done by mixing equal amounts (150 ng) of each Entry plasmid and a single Destination vector with 2 μL of LR Clonase II Plus enzyme mix (Invitrogen, Cat # 12538120) and TE in a total reaction volume of 10 μL and incubated overnight at room temperature. The volume of these reactions may be reduced by half (to 5 μL). Please note that the manufacturer suggests combining 20 femtomoles of expression vector and between 10-20 femtomoles of each entry clone. Calculators are available online to account for mass to ensure the proper molar ratios are followed (Mosimann, 2022). The following day, the reaction was terminated by the addition of 1 μL of Proteinase K (2 μg/μL) and incubated at 37ºC for 10 minutes, followed by transformation into chemically competent STBL3 bacteria (Invitrogen, Cat #C737303) and plating on LB agar plates containing the appropriate antibiotic (in most cases ampicillin,

unless otherwise noted). Clones were screened by colony PCR using primers that flanked the insert(s). All positive clones were then confirmed by Sanger sequencing across each *att* recombination site or nanopore whole plasmid sequencing (i.e. Plasmidsaurus).

pDEST-EF1a-EGFP-mm-miR-500 (JDW 1477), pDEST-EF1a-EGFP-miR-MCS (JDW 1478) and pDEST-EF1a-EGFP-hs-miR-126 (JDW 1415) were made by performing an LR Cloning reaction between pME-EGFP-mm-miR-500 (JDW 8), pME-EGFP-hs-miR-126 (JDW 1401), or pME-EGFP-miR-MCS (JDW 1231), and pDEST-EF1a (Invitrogen).

pCAGEN-rtTA-IRES-nls-EGFP-WPRE-bGH-pA (JDW 410) was created by an LR reaction between pME-rtTA-IRES-3xNLS-EGFP-WPRE-bGH-pA (JDW 484) and pCAGEN-DEST (JDW 471).

pCAGEN-nls-BFP-FLAG-WPRE-pA (JDW 1500) was created by performing an LR reaction between pME-3xNLS-mTagBFP-FLAG-bGH-pA (JDW 484) and pCAGEN-DEST (JDW 471).

To generate pCS-Phi31o (mouse codon optimized, phage Phi31 integrase) (JDW 453), Phi31o (codon optimized) was amplified from Addgene (#13795, pPGKPhiC31obpA, a gift of Dr. Phillip Soriano)(Raymond and Soriano, 2007) to generate pME-Phi31o. This construct was recombined into a pCS2-DEST (Addgene # 22423, a kind gift from Dr. Nathan Lawson, University of Massachusetts Medical School). pCS-Phi31o can be linearized via NotI digestion and used for in vitro transcription to generate capped, stable mRNA for co-injection with plasmids containing attB sites into mouse oocytes or zebrafish embryos that contain attP landing sites in their genome for stable integration, as in (Devine et al., 2014, Lalonde et al., 2024).

To generate pAAV-Tet-Off-FLEx-Lifeact-mScarlet-HA-WPRE-pA (JDW 721), Lifeact-mScarlet-HA was amplified using oJDW 1035 5’-ATATCTCTCGAGGGCGCGCCTTAAGCGTAATCTGGAACGTCATATGGATACTTGTACAGCTCGTCCATGCCGCCGGTGGAGTG and oJDW 1038 5’-ATATCTGCTAGCGCCACCATGGGCGTGGCCGACCTGATCAAGAAGTTCGAGAGCATCAG. The resulting amplicon was digested with NheI and AscI and was inserted into JDW 712 (pAAV-Tet-Off-FLEX-MCS-WPRE-pA) via the same enzymes.

An LR reaction between pME-Luciferase-P2A-H2A-mCherry (JDW 926) and pB-Tet-On-DEST-EFS-mODC-rtTA-IRES-NEO (JDW 931) was used to generate pB-Tet-On-Luciferase-P2A-H2A-mCherry-IRES-NEO (JDW 945). An LR reaction between pME-Luciferase-P2A-H2A-mCherry (JDW 926) and pB-Tet-Off-FLEX-EFS-d2tTA (JDW 936) to generate pB-Tet-Off-FLEX-Luciferase-P2A-H2A-mCherry (JDW 970). An LR reaction between pME-Luciferase-P2A-H2A-mCherry (JDW 926) and pCAGEN-Dest (JDW 471) was used to generate pCAGEN-Luciferase-P2A-H2A-mCherry (JDW 1129). pB-Tet-On-DEST-hEF1a-mODC-rtTA-IRES-NEO (JDW 1086) was used in an LR reaction with pME-

Luciferase-P2A-H2A-mCherry (JDW 926) to generate pB-Tet-On-Luciferase-P2A-H2A-mCherry-hEF1a-mODC-rtTA-IRES-NEO (JDW 1130), while JDW 926 was recombined with pB-TA-ERN (Addgene # 80474) to generate pB-Tet-On-Luciferase-P2A-H2A-mCherry-CMVie-rEF1a-rtTA-ires-NEO (JDW 1154).

pB-CAG-lsl-mScarlet-V5 (JDW 1404) was generated by multisite LR recombination between p5E-CAG (JDW 912), pME-Lox-3xStop-Lox (JDW 869), p3E-V5-mScarlet-stop-pA (JDW 968), and pB-DEST (JDW 1205).

pCAGEN-Dre-nls (JDW 1131) and pCAGEN-DreERT2 (JDW 486) were generated by an LR reaction between pME-Dre-nls (JDW 31) or pME-DreERT2 (JDW 1344) and pCAGEN-DEST (JDW 471). pMEF2c-AHF-Dre-nls (JDW 35) and pMEF2c-AHF-DreERT2 (JDW 196) were generated by LR recombination between pME-Dre-nls (JDW 31) or pME-DreERT2 (JDW 1344) and pMEF2c-AHF-DEST (JDW 478).

pCAGGS-rox-nKateV5-DEST-FRT-NEO (JDW 472) was recombined with pME-myr-BFP-Flag (JDW 1183) in an LR reaction to generate pCAGGS-nK-mB (aka pCAGGS-*rox*-nKateV5-*rox*-myrBFPFlag-FRT-PGK-NEO-FRT (JDW 473). The entire cassette can be excised via AscI and PacI digestion for subsequent cloning to a targeting vector, as done for the *Hipp11* locus (*pHipp11-rox*-nK-*rox*-mB-FRT-NEO, JDW 479) and the *Rosa26* locus (Devine et al., 2014).

pCS2-Sun1-2xsfGFP-6xMyc-pA (JDW 694) was created by performing an LR Cloning reaction between JDW 681 (pME_Sun1_2xsfGFP_6xMyc_polyA) and pCS2-pDEST (Addgene 22423)(Villefranc et al., 2007).

pCAGEN-Vhh-Lamin-mNeonGreen-myr-BFP-SV40pA (JDW 1353) was generated by an LR reaction between pME-Vhh-LaminB-mNeonGreen (JDW 1312) and pCAGEN-DV-IRES-myr-BFP (JDW 491). Due to the presence of the SV40polyA 5’ to the IRES the myrBFP reporter is not expected to be generated.

pCAGEN-Actin-Vhh-mNeonGreen-HA-pA (JDW 1308) was generated by an LR reaction between pME-Actin-Vhh-mNeonGreen-HA-pA (JDW 1248) and pCAGEN-DV (JDW 471).

pCAGEN-nls-mCherry-IRES-myr-EGFP (JDW 1326) was generated by an LR reaction between pME-nls-mCherry-stop (JDW 418) and pCAGEN-DEST-IRES-myr-EGFP (JDW 495).

pTol2-Hsp70-lox-H2B-mCerulean-stop-lox-mScarlet; fli1ep-zCreI-BFP (JDW 1233) was generated by an LR reaction (p5E-Hsp701) (JDW 458 / Chien Lab #222)(Kwan et al., 2007), pME-loxP-H2B-mCerulean-2xSTOP-loxP (a kind gift of Drs. Michael Harrison and Ellen Lien at USC)(Harrison et al., 2015), p3E V5-mScarlet-I (JDW 461), and pTol2-Dest-fli1ep-zCreI-BFP (JDW 1218).

Development • Supplementary information

pTol2-fli1ep-lox-AmCyan-stop-lox-mScarlet; hsp70-zCreI-BFP (JDW 1234) was made by multisite LR recombination between p5E_Fli1ep (p5Efli1ep) (Lawson lab #478)(Villefranc et al., 2007), pME-loxP-AmCyanSTOP-lox (a kind gift of Drs. Caroline and Geoff Burns at Boston Children's Hospital)(Zhou et al., 2011), p3E-V5-mScarlet-I-stop-Sv40pA (JDW 968) and pTol2-Dest-hsp70-zCreI-BFP (JDW 1184).

pTol2-Ubi-lox-EGFP-stop-lox-nls-mCherry; hsp70-zCreI-BFP (JDW 1235) was made by multisite LR recombination between pENTR5-ubi-loxP-EGFP-loxP (Addgene # 27322), pME-nls-mCherry-stop (Chien lab #233)(Kwan et al., 2007), p3E-polyA (Chien Lab plasmid #302) (Kwan et al., 2007)and pTol2-Dest-hsp70-zCreI-BFP (JDW 1184).

pTol2-Ubi-lox-EGFP-stop-lox-nls-mCherry; hsp70-zCreI-mTagBFP-mODC-2xIns (JDW 1057) was generated by multisite LR recombination between pENTR5'-ubi:loxP-EGFP-loxP (Addgene # 27322), pME-nls-mCherry-stop (Chien lab #233), p3E-polyA (Chien Lab plasmid #302) and pTol2-Hsp70-zCreI-mTagBFP2-mODC-2xIns (JDW 1002).

pTol2-Ubi-lox-EGFP-stop-lox-nls-mCherry; fli1ep-zCreI-BFP (JDW 1236) was created by performing a multisite LR reaction with pENTR5-ubi-loxP-EGFP-loxP (Addgene # 27322), pME-nls-mCherry-stop (Chien lab #233), p3E-polyA (Chien Lab plasmid #302) and JDW 1218 (pTol2-Dest-zFli1-zCreI-BFP).

pTol2-Ubi-lexA-lexO-nls-GFP-pA (JDW 1316) was created by performing a multisite LR reaction between p5E-Ubi-pro (JDW 804, which we created by digest of Addgene #27322 via BamHI digestion and ligation, but is identical to Addgene # #27320)(Mosimann et al., 2011), pME-lexPR (a kind gift of Dr. Holger Gerhardt, Max-Delbrück-Centrum für Molekulare Medizin), p3E-nls-GFP-pA (Addgene #80814, a kind gift of Dr. Philip Washbourne's lab, University of Oregon) and pDEST-Tol2-CG2 (Chien lab plasmid #395).

pTol2-Ubi-dLexA-lexO-nls-GFP-pA (JDW 1381) was generated by performing a multisite LR reaction between p5E-Ubi-pro (JDW 804, which we created by digest of Addgene #27322 via BamHI digestion and ligation, but is identical to Addgene # #27320), pME-LexA-mODC-LexOp-35SCaMV (JDW 1348), p3E-nls-GFP-pA (Addgene #80814, a kind gift of Dr. Philip Washbourne's lab, University of Oregon) and pDEST-Tol2-CG2 (Chien lab plasmid #395).

pTol2-Ubi-dLexA-lexO-c-fos-nls-GFP-pA (JDW 1382) was generated by performing a multisite LR reaction between p5E-Ubi-pro (JDW 804, which we created by digest of Addgene #27322 via BamHI digestion and ligation, but is identical to Addgene # #27320), pME-LexA-mODC-LexAOp-cFos (JDW 1359), p3E-nls-GFP-pA (Fowler et al., 2016)(Addgene #80814, a kind gift of Dr. Philip Washbourne's lab, University of Oregon) and pDEST-Tol2-CG2 (Chien lab plasmid #395)(Kwan et al., 2007).

Development • Supplementary information

pCAGEN-FastFUCCI-P2A-H2A-tdiRFP (JDW 1402) was created by performing an LR recombination between pME-FUCCI-P2A-H2A-tdiRFP (JDW 1399) and pCAGEN-DV (JDW 471).

pCAGEN-FastFUCCI (JDW 1494) was created by performing an LR recombination between pME-FastFucci (JDW 1488) and pCAGEN-DV (JDW 471).

pCAGEN-FastFUCCI-IRES-myr-mTagBFP (JDW 1502) was created by performing an LR recombination reaction between pME-FastFUCCI (JDW 1488) and pCAGEN-DV-IRES-myr-BFP (JDW 491).

pCAGEN-FastFUCCI-IRES-myr-mKate2 (JDW 1503) was created by performing an LR recombination reaction between pME-FastFUCCI (JDW 1488) and pCAGEN-DV-IRES-myr-mKate2 (JDW 494).

pCAGEN-H2B-V5-mScarlet3-S2 (JDW 1529) was generated by performing an LR recombination reaction between pME-H2B-V5-mScarlet3-S2 (JDW 1514) and pCAGEN-DV (JDW 471).

pCAGEN-H2B-V5-mScarlet3-H (JDW 1530) was generated by performing an LR recombination reaction between pME-H2B-V5-mScarlet3-H (JDW 1513) and pCAGEN-DV (JDW 471).

pCAGEN-ActinVhh-sfGFP-P2A-HA-tdiRFP-caax (JDW 1532) was generated by performing an LR recombination reaction between pME-ActinVhh-sfGFP-P2A-HA-tdiRFP-caax (JDW 1311) and pCAGEN-DV (JDW 471).

pB-EF1a-FastFUCCI-IRES-3xnls-mTagBFP2 (JDW 1511) was created by performing a multisite LR reaction between p5E-hsEF1a (JDW 1164), pME-FastFUCCI (JDW 1488), p3E-IRES-3xnls-mTagBFP2 (JDW 1361), and pB-DEST (JDW 1205).

pB-EFS-FastFUCCI-IRES-3xnls-mTagBFP2 (JDW 1515) was created by performing a multisite LR reaction between p5E-hsEFS (JDW 1319), pME-FastFUCCI (JDW 1488), p3E-IRES-3xnls-mTagBFP2 (JDW 1361), and pB-DEST (JDW 1205).

pTol2-Ubi-zFUCCI-pA; exorh-GFP (JDW 1489) was generated by performing a multisite LR reaction between p5E-Ubi-pro (JDW 804, which we created by digest of Addgene #27322 via BamHI digestion and ligation, but is identical to Addgene # #27320), pME-zFUCCI (JDW 1464), p3E-polyA (Chien Lab plasmid #302), and pDest exorh-EGFP (Addgene #195983, a kind gift of Dr. Christian Mosimann, University of Colorado School of Medicine).

pTol2-Crestin-zFUCCI-IRES-EGFP-caax (JDW 1516) was generated by performing a multisite LR reaction between p5E-Crestin (JDW 1320), pME-zFUCCI (JDW 1464), p3E-IRES-EGFP-caax (Chien lab plasmid #389), and pDEST-Tol2-pA (Chien Lab plasmid #394), both kind gifts of the late Dr. Chi-Bin Chien (University of Utah)(Kwan et al., 2007).

Development • Supplementary information

pTol2-Fli1-zFUCCI-IRES-mTagBFP2 (JDW 1517) was generated by performing a multisite LR reaction between p5E_Fli1ep (p5Efli1ep) (Lawson lab #478)(Villefranc et al., 2007), pME-zFUCCI (JDW 1464), p3E-IRES-mTagBFP2-pA (JDW 1361), and pDEST-Tol2-pA (Chien Lab plasmid #394) (Kwan et al., 2007).

pTol2-Unc-Actin-mCherry-IRES-StayGold-pA (JDW 1528) was generated by performing a multisite LR reaction between p5E-Unc-cFos-b-globin (JDW 1365), pME-Actin-Vhh-mCherry (JDW 1240), p3E-IRES-StayGold-pA (JDW 1364), and pDEST-Tol2-pA (Chien Lab plasmid #394).

pLenti-BLAST-CMV-LiCre-WPRE (JDW 1495) was created by performing an LR reaction with pME-LiCre (JDW 1119) and pLenti-CMV-Blast-DEST (Addgene #17451) (Campeau et al., 2009).

pCAGEN-hyPBase-WPRE (JDW 1369) was created by performing an LR recombination between pME-hyPBase-WPRE (JDW 679) and pCAGEN-DEST (JDW 471). Note, that due to MTA issues with the patent holder for *piggyBac* technology, Hera BioLabs, this construct cannot be distributed by Addgene.

pGlast-hyPBase-WPRE (JDW 1166) was created by performing an LR reaction between pME-hyPBase-WPRE (JDW 679) and pGLAST-DEST (JDW 1009). Note, that due to MTA issues with the patent holder for *piggyBac* technology, Hera BioLabs, this construct cannot be distributed by Addgene.
